# Post COVID-19 syndrome among 5248 healthcare workers in England: longitudinal findings from NHS CHECK

**DOI:** 10.1136/oemed-2024-109621

**Published:** 2024-10-02

**Authors:** Brendan Dempsey, Helen A Blake, Ira Madan, Sharon A M Stevelink, Neil Greenberg, Rosalind Raine, Anne-Marie Rafferty, Rupa Bhundia, Simon Wessely, Danielle Lamb

**Affiliations:** 1 Department of Primary Care and Population Health, University College London, London, UK; 2 Guys and St Thomas NHS Foundation Trust, London, UK; 3 Institute of Psychiatry, Psychology & Neuroscience, King's College London, London, UK; 4 Florence Nightingale Faculty of Nursing, Midwifery and Palliative Care, King's College London, London, UK

**Keywords:** COVID-19, Epidemiology, Health Personnel, Longitudinal studies

## Abstract

**Objectives:**

The objectives of this study were to examine post COVID-19 syndrome (PCS) among healthcare workers (HCWs) in England and explore risk factors for the condition.

**Methods:**

Data were collected by National Health Service (NHS) CHECK, a longitudinal study exploring HCWs’ mental and physical well-being during and after the COVID-19 pandemic. NHS CHECK collected data at four timepoints: the baseline survey between April 2020 and January 2021, and then three follow-up surveys at approximately 6, 12 and 32 months post baseline. PCS data were collected at 12 and 32 months, while risk factor data were from baseline. HCWs were asked what COVID-19 symptoms they experienced and for how long and were classified as having PCS if they had any symptom for ≥12 weeks. Multilevel regressions were used to examine risk factors for PCS.

**Results:**

This study included 5248 HCWs. While 33.6% (n=1730) reported prolonged COVID-19 symptoms consistent with PCS, only 7.4% (n=385) reported a formal diagnosis of PCS. Fatigue, difficult concentrating, insomnia and anxiety or depression were the most common PCS symptoms. Baseline risk factors for reporting PCS included screening for common mental disorders, direct contact with COVID-19 patients, pre-existing respiratory illnesses, female sex and older age.

**Conclusions:**

While a third of HCWs reported prolonged COVID-19 symptoms consistent with PCS, a smaller percentage reported a formal diagnosis of the condition. We replicate findings that direct contact with COVID-19 patients, older age, female sex, pre-existing respiratory illness and symptoms of common mental disorders are associated with increased risk of PCS.

**WHAT IS ALREADY KNOWN ON THIS TOPIC**

- The pathogenesis of post COVID-19 syndrome (PCS) remains unknown, though suggested risk factors may include female sex, older age, pre-existing respiratory illnesses and preinfection mental disorders.

**WHAT THIS STUDY ADDS**

- While one-third of people in this sample with a previous COVID-19 infection reported symptoms lasting for ≥12 weeks, indicating PCS, only 7.4% reported having been formally diagnosed with PCS.
- Screening positive for mental disorders was the strongest observed risk factor for reporting PCS at least 12 months later. Direct contact with COVID-19 patients, having a pre-existing respiratory illness, female sex and older age were also risk factors.

**HOW THIS STUDY MIGHT AFFECT RESEARCH, PRACTICE OR POLICY**

- Though PCS symptoms were prevalent, more work is needed to understand their prevalence in the general population in order to understand the specific impact of COVID-19 on their reporting.
- Failure to assess mental disorders as a risk factor for PCS or Long COVID, along with other important factors, will weaken our understanding of the condition.

## Introduction

 Long COVID (LC) occurs when COVID-19 symptoms persist or continue to develop after the acute infection.^1 2^ The National Institute for health and Care Excellence (NICE) differentiates between ongoing symptomatic COVID-19 (symptoms between 4 and 12 weeks) and post-COVID-19 syndrome (PCS; symptoms ≥12 weeks).^1^ LC was poorly understood early in the pandemic,^3 4^ and consensus on pathogenesis or prognosis remains elusive.^5 6^ Inflammation may be responsible, as a recent study found that among people hospitalised during the acute COVID-19 infection, prolonged symptoms were associated with inflammation of myeloid cells and activation of the complement system.^7^ The UK Office for National Statistics (ONS) estimates that the most common symptoms include fatigue (reported by 72% of those with PCS), difficulty concentrating (51%), muscle ache (49%) and shortness of breath (48%).^8^ Further, 59% of people with PCS reported that their symptoms had impacted their day-to-day activities ‘a little’, while 20% said they had been ‘limited a lot’.^8^

PCS prevalences vary. A meta-analysis found that prevalence of symptoms lasting ≥12 weeks among people with confirmed or probable COVID-19 infections was lower in studies using healthcare records to diagnose caseness (13.6%; 95% CI 1.2% to 68.0%) compared with studies using self-reported symptoms (43.9%; 95% CI 8.2% to 87.2%).^9^ The ONS estimated that approximately 1.7 million people in the UK had self-reported PCS in February 2023 (~3% of the population and ~9% of all PCR-confirmed COVID-19 infections).^8^ While the mechanisms of PCS remain unknown, a meta-analysis identified risk factors, including female sex, older age, having a pre-existing respiratory illness, being unvaccinated against COVID-19 and being hospitalised during the acute COVID-19 infection.^10^ History of common mental disorders (CMDs) prior to COVID-19 infection has also been explored, though this remains under-researched and poorly understood.^10 11^

Healthcare workers (HCWs) were at high risk to COVID-19 infection.^12^ In the UK, research using data from the ONS COVID-19 Infection Survey found that HCWs were at higher risk of contracting COVID-19 compared with non-essential workers, though this effect reversed by June 2021.^13^ Another study found that HCWs were among the occupational groups to self-report higher prevalences of COVID-19 symptoms lasting 4 or more weeks.^14^ ONS mortality data revealed that those working in the healthcare sector were more likely to die of COVID-19 in 2020 compared with other industries, with this effect diminishing thereafter.^15^ Given the increased risk of COVID-19 infection among HCWs, particularly early in the pandemic, they are likely to be at high risk of PCS. This may also be the case for other essential workers, with educators, bus and coach drivers, and police and protective services staff also at increased risk.^12 13^

Due to the associated symptoms and lack of a known mechanism, PCS has drawn comparisons with other conditions, such as myalgic encephalomyelitis/chronic fatigue syndrome (ME/CFS).^5^ ME/CFS is a long-term condition defined by postexertional malaise, which may have a delayed onset, physical and mental fatigue and fatigability, issues with sleeping, memory or concentration, and functional impairment, which may be substantial.^16^ Diagnoses are made if the defining symptoms last for 6 months or longer and cannot be attributed to an alternative cause.^16^ Risk factors include female sex, older age, having asthma and a history of CMDs.^17 18^

We examined the prevalence, symptoms and risk factors for PCS among a large sample of HCWs in England. Given the paucity of longitudinal evidence in this area, we explored whether factors identified in previous longitudinal post infectious fatigue syndrome work,^17 18^ such as female sex, older age and symptoms of CMDs, would be significant risk factors for reporting PCS.

## Methods

### NHS CHECK and data collection

We analysed data from NHS CHECK, a longitudinal survey distributed to all HCWs (clinical and non-clinical), students and volunteers in 18 NHS Trusts across England at four data collection periods from April 2020 to May 2023 (baseline, then 6, 12 and 32 months post baseline). Trusts were invited to distribute an online survey (paper copies available on request) via direct emails to senior leadership teams and were purposively selected to offer diversity in geographical location, urban and rural settings, and acute and mental health Trusts. Trusts promoted the survey to all eligible staff via existing group emails. We also promoted the survey via staff support teams/leads, chief nursing officers, medical directors, occupational health departments, trade union representatives and well-being hub users. NHS CHECK was discussed during team briefings, advertised via screen savers on Trust computers and included in Trust newsletters, news items on Trust intranet websites and closed social media groups. At baseline, participants completed a short survey and were given the option to complete an additional longer survey. To account for attrition, a replenishment sample of HCWs who did not complete the baseline or 6-month surveys was recruited at 12 months. The NHS CHECK cohort consists of 24 137 participants. For further information, see the cohort protocol^19^ and this study’s preregistered protocol.^20^ We followed the Strengthening the Reporting of Observational Studies in Epidemiology guideline for cohort studies for this report.^21^

### Outcome

Data on PCS were collected at 12 and 32 months. At 12 months, staff were asked if they had ever been diagnosed with COVID-19. Those who had were given a list of 24 symptoms and asked to report whether they had developed each symptom due to COVID-19 and for how long they experienced it using a 4-point scale (1 day–2 weeks, 2–4 weeks, 4–12 weeks, ≥12 weeks). These symptoms were chosen based on NICE guidance at the time of data collection.^1^ At 32 months, we asked staff the same questions but only regarding COVID-19 infections within the preceding year, ensuring two unique measurements. We also asked staff to self-report if they had received a formal diagnosis of LC or PCS from a medical professional. Following NICE guidance, we defined staff as having PCS if they experienced any symptom for ≥12 weeks, regardless of whether they had recovered by the time of the study or had been formally diagnosed with LC/PCS.^1^ We created a binary outcome variable to represent caseness (0=did not report PCS, 1=reported PCS).

### Risk factors

Variables were included if they were risk factors in the LC^10^ or the ME/CFS literature,^17 18^ as well as additional variables that were available in the NHS CHECK cohort.

#### Mental disorder variables

Our primary mental disorder variable was CMDs (measured by the General Health Questionnaire (GHQ-12^22^)). The robustness of the GHQ-12 measure was established even where response options differ slightly from the original, as was the case in this study where there was a small typographical error in one response option of one GHQ-12 item.^23 24^ We included five other mental disorder variables, but due to multicollinearity and higher levels of missing data, these were considered secondary variables and were examined in sensitivity analysis only: depression (Patient Health Questionnaire (PHQ-9^25^)), generalised anxiety disorder (GAD-7^26^), burnout (Burnout Assessment Tool (BAT-12^27^)), post-traumatic stress disorder (PTSD; Post-traumatic stress disorder Check List (PCL-6^28^)) and alcohol use disorder (Alcohol Use Disorder Identification Test for Consumption (AUDIT-C^29^)). Using the recommended cut-off scores, we screened for probable mental disorders: GHQ-12 scores ≥4; PHQ-9 scores≥10, GAD-7 scores ≥10, BAT-12 scores ≥2.96, PCL-6 scores ≥14 and AUDIT-C scores ≥8. Alcohol-use disorder did not violate multicollinearity and was included in all models.

#### Covariates

Covariates included participants’ age, sex, ethnicity, job role, relationship status, self-reported pre-existing respiratory illnesses (ie, asthma or chronic obstructive pulmonary disease (COPD)), contact with COVID-19 patients, income, perceived access to personal protective equipment (PPE) and confidence in workplace infection control policies. All covariates were measured in the short baseline survey.

### Analyses

We preregistered an analysis protocol on the Open Science Framework,^20^ and this paper addresses research questions 1, 2 and 3. Our sample was adequately powered. The cohort was weighted using a raking algorithm based on the age, sex, ethnicity and job role profile of the workforce at each Trust to maximise representativeness.^19^ To complete the weighting, missing data were imputed using the fifth nearest neighbour algorithm. These imputed data were only used to complete weighting. Weighting was conducted using R V.4.0.2. All percentages in the results were weighted.

**Figure 1:**
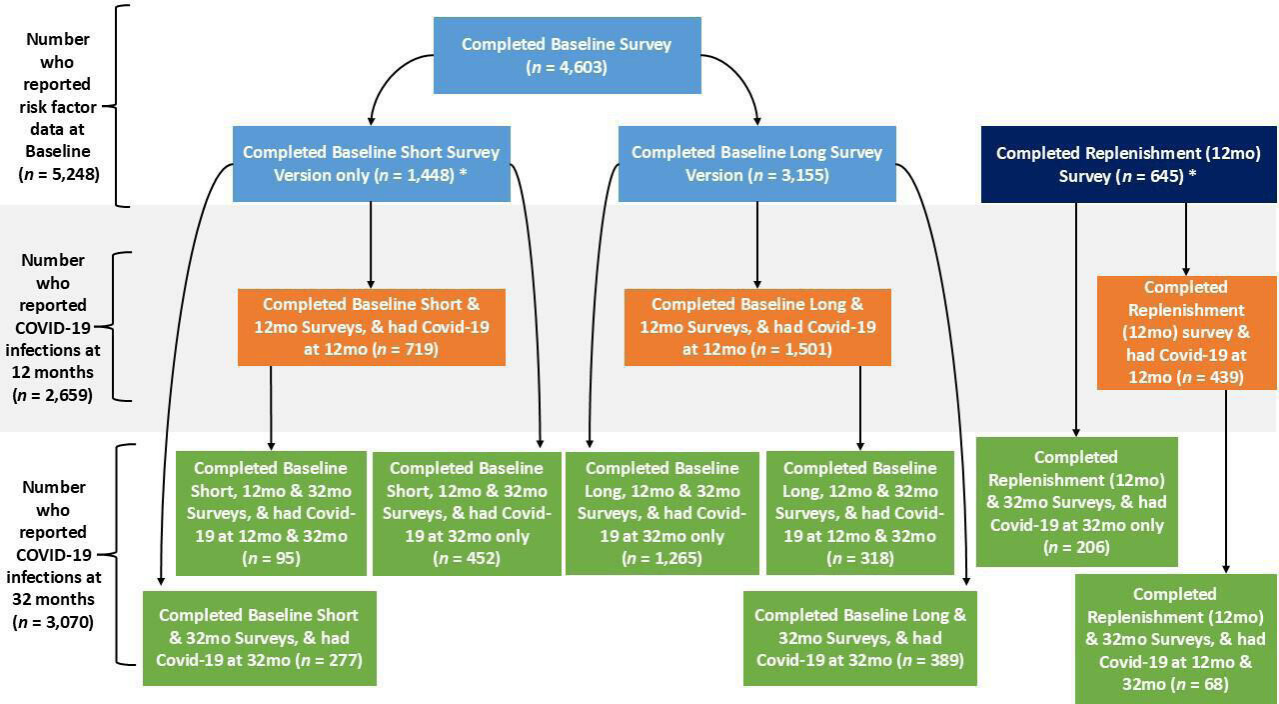
Participation in each National Health Service (NHS) CHECK survey wave for healthcare workers with a previous COVID-19 infection. 12mo=12 month follow-up survey; 32mo=32 month follow-up survey. *These 2093 participants (39.3% of all those who reported a previous COVID-19 infection) do not have complete scores for the secondary mental disorder variables as they were only included in the longer baseline survey.

As per our protocol, analysis began with descriptive statistics on the baseline risk factors, outcome variable and each COVID-19 symptom and its duration. To account for missing risk factors data and maintain power, we conducted Multiple Imputation using Chained Equations (MICE). See section A of the online supplemental materials for a detailed description of the MICE procedure (including online supplemental tables 1a,b and 2 and 3a,b and onlinesupplemental figures 1  2).

The imputed datasets were then pooled using Rubin’s rules and included within a multilevel logistic regression to examine risk factors. The model was multilevel as we grouped participants by Trust to account for clustering. In addition, we included an exposure variable. We identified ‘burden periods’, that is, periods of time at baseline when the reported number COVID-19 cases and deaths were higher or lower, which likely influenced pressure on HCWs and may have influenced the risk factors, particularly the mental disorder variables.^19^ Reported ORs are adjusted for all variables in the model. For post-hoc sensitivity analyses, we reran the regression model with a number of changes: using only formal diagnosis of LC/PCS as the outcome; using the broad NICE definition of LC (≥4 weeks of symptoms) as the outcome^1^; using only data collected at 12 or 32 months; and with the secondary mental disorder variables. Data analysis was completed using Stata V.18.

### Patient and public involvement and engagement

A patient and public involvement and engagement (PPIE) group was set up as part of this study. The group comprised 16 HCWs with experience of PCS from a range of demographic and professional backgrounds. The group met several times to discuss the project aims, analysis plans and the findings.

## Results

### Sample characteristics

5248 HCWs reported a previous COVID-19 infection (22.5% of the full NHC CHECK cohort and 45.9% of the 11 776 HCWs who responded to this question at 12 or 32 months). As this study uses data from three waves of data collection, we examined participation across each (figure 1). Most HCWs (88.1%) participated at baseline, with 11.9% joining at 12 months. Of those who completed the baseline survey, most (n=3155; 68.9%) completed the longer survey. At 12 months, 2659 HCWs reported a previous COVID-19 infection, while 3070 reported a COVID-19 infection at 32 months; 481 HCWs reported a previous COVID-19 infection on both.

Table 1 shows the demographic and occupational information for the HCWs. Most were female, white and in a relationship. Approximately 8% of those who reported a previous COVID-19 infection were doctors, and the remainder were roughly evenly split between nursing, other clinical and non-clinical staff. Most did not have asthma or COPD. The majority had direct contact with COVID-19 patients and perceived good access to PPE, but 35.1% of all HCWs with a previous COVID-19 infection reported inadeuqate workplace infection control policies. Most completed the baseline survey prior to testing positive for a COVID-19 infection. Table 2 shows information on probable mental disorders among the HCWs’. CMDs were the scale with the highest percentage of staff meeting the cut-off score (ie, above the threshold), followed by depression, PTSD, then GAD. See online supplemental table 4a–d for information about the sample at the 12-month and 32-month surveys.

**Table 1:** Baseline demographic and occupational information of the healthcare workers in NHS CHECK

Variable	Total NHS CHECK sample	Staff with a previous COVID-19 infection	Staff with PCS
	n=24 137 (%)	n=5248 (%)	n=1730 (%)
Sex			
Female	19 381 (74.1)	4234 (74.7)	1451 (78.5)
Male	4491 (24.9)	973 (24.5)	267 (21.0)
Missing	265 (1.0)	41 (0.8)	12 (0.5)
Age in years			
30 and younger	4681 (21.3)	754 (16.2)	220 (13.8)
31–40	5247 (25.0)	1010 (22.8)	304 (21.2)
41–50	6000 (22.0)	1457 (24.3)	500 (24.8)
51–60	5691 (20.7)	1500 (25.9)	529 (29.4)
61 and older	1439 (6.5)	321 (7.0)	102 (6.3)
Missing	1079 (4.5)	206 (3.8)	75 (4.5)
Ethnicity			
White	20 507 (75.9)	4665 (81.2)	1549 (83.0)
Black	1045 (8.0)	155 (6.3)	41 (5.3)
Asian	1572 (12.1)	242 (9.0)	72 (8.1)
Mixed/multiple ethnic group	593 (1.1)	125 (1.2)	47 (1.2)
Other ethnic group	224 (2.2)	34 (1.9)	13 (2.1)
Missing	196 (0.7)	27 (0.4)	8 (0.3)
Job role			
Nurse	6127 (29.7)	1376 (30.7)	528 (34.9)
Doctor	1727 (9.8)	340 (8.3)	81 (5.8)
Other clinical	7338 (32.1)	1583 (32.3)	522 (32.1)
Non-clinical	8744 (27.7)	1910 (28.1)	589 (26.7)
Missing	201 (0.7)	39 (0.6)	10 (0.5)
Relationship status			
Single/divorced	6304 (26.9)	1259 (24.3)	409 (24.7)
Married/cohabitating	17 592 (72.2)	3958 (75.2)	1311 (74.9)
Missing	241 (0.9)	31 (0.5)	10 (0.4)
Pre-existing respiratory illness*			
None	21 338 (89.1)	4295 (82.9)	1378 (81.1)
Reported asthma/COPD	1216 (5.0)	308 (5.2)	139 (7.0)
Missing	1583 (5.9)	645 (11.9)	213 (11.9)
Income			
NHS Band 5 or below	7599 (27.7)	1643 (27.0)	570 (28.9)
NHS Band 6 or above	12 570 (54.0)	2814 (57.0)	931 (57.6)
Missing	3968 (18.3)	791 (16.0)	229 (13.5)
Contact with COVID-19 patients*			
No contact	8200 (27.1)	1526 (22.3)	360 (17.4)
Contact	10 309 (51.7)	2325 (52.6)	891 (56.7)
Missing	5628 (21.2)	1397 (25.1)	479 (25.9)
Perceived access to personal protective equipment*			
Perceived inadequate access	1709 (8.3)	351 (7.9)	147 (10.1)
Perceived adequate access	17 130 (73.8)	3566 (69.2)	1166 (67.2)
Non-applicable	2839 (8.6)	564 (8.7)	158 (8.2)
Missing	2459 (9.3)	767 (14.2)	269 (14.5)
Confidence in infection control policies*			
Inadequate confidence	8211 (37.7)	1656 (35.1)	598 (36.5)
Adequate confidence	13 406 (53.0)	2824 (50.9)	885 (49.5)
Missing	2520 (9.3)	768 (14.0)	247 (14.0)
COVID-19 testing status at time of baseline survey response*			
Tested positive for COVID-19	1254 (7.2)	528 (13.0)	280 (17.6)
Tested negative for COVID-19	7362 (32.0)	1457 (28.5)	455 (26.5)
Tested, awaiting result	237 (1.0)	43 (0.8)	23 (1.2)
Tested, inconclusive result	163 (0.7)	44 (0.9)	24 (1.5)
Never tested	12 612 (49.7)	2416 (42.6)	697 (39.0)
Missing	2509 (9.4)	760 (14.2)	251 (14.2)

n = unweighted frequencies (with weighted percentages). NHS Band 5 or below = annual income of £34 581 or lower; NHS Band 6 or above = annual income of £35 392 or higher (as of January 2024). Data for all variables were collected at baseline, between April 2020 and January 2021 and will be used to explore risk factors for reporting PCS symptoms, with the expection of COVID-19 testing status.

* These questions were not asked to the replenishment sample, accounting for 1583 missing responses from the total NHS CHECK sample, 645 from staff with a previous COVID-19 infection and 213 from staff with PCS.

COPDchronic obstructive pulmonary diseaseNHSNational Health Service in the UKPCSpost COVID-19 syndrome (symptoms lasting for 12 weeks following acute COVID-19 infection)

**Table 2:** Baseline information on probable mental disorders among healthcare workers in NHS CHECK

Variable	Total NHS CHECK sample	Staff with a previous COVID-19 infection	Staff with PCS
	n=24 137 (%)	n=5248 (%)	n=1730 (%)
Probable common mental disorders			
No (GHQ-12 score <4)	9811 (40.9)	1959 (36.7)	501 (27.3)
Yes (GHQ-12 score ≥4)	10 970 (45.9)	2382 (46.2)	918 (54.9)
Missing	3356 (13.2)	907 (17.1)	311 (17.8)
Probable depression*			
No (PHQ-9 score <10)	8694 (35.3)	2140 (40.7)	620 (36.3)
Yes (PHQ-9 score ≥10)	3302 (14.3)	890 (17.6)	400 (24.0)
Missing	12 141 (50.4)	2218 (41.7)	710 (39.7)
Probable generalised anxiety disorder*			
No (GAD-7 score <10)	9319 (38.0)	2372 (45.2)	736 (43.1)
Yes (GAD-7 score ≥10)	2716 (11.8)	673 (13.4)	293 (17.8)
Missing	12 102 (50.2)	2203 (41.4)	701 (39.1)
Probable burnout*			
No (BAT-12 score <2.96)	10 000 (41.1)	2512 (48.1)	798 (46.4)
Yes (BAT-12 score ≥2.96)	1820 (7.7)	486 (9.5)	212 (13.1)
Missing	12 317 (51.2)	2250 (42.4)	720 (40.5)
Probable post-traumatic stress disorder*			
No (PCL-6 score <14)	9158 (36.9)	2303 (43.2)	699 (39.6)
Yes (PCL-6 score ≥14)	2825 (12.6)	730 (14.9)	319 (20.3)
Missing	12 154 (50.5)	2215 (41.9)	712 (40.1)
Probable alcohol use disorder*			
No (AUDIT-C score <8)	10 010 (42.0)	2534 (49.0)	854 (51.0)
Yes (AUDIT-C score ≥8)	1396 (5.3)	337 (6.2)	110 (6.0)
Missing	12 731 (52.7)	2377 (44.8)	766 (43.0)

n = unweighted frequencies (with weighted percentages). PCS = Post COVID-19 Syndrome (symptoms lasting for 12 weeks following acute COVID-19 infection).

Data for all variables were collected at baseline, between April 2020 and January 2021, and will be used to explore risk factors for reporting PCS symptoms.

* These were only completed by those who took part in the longer baseline survey (3155 of 5248 HCWs; 60.7%), hence have a higher percentage of missing data.

AUDIT-CAlcohol Use Disorder Identification Test for ConsumptionBATBurnout Assessment ToolGADGeneralised Anxiety DisorderGHQGeneral Health QuestionnaireNHSNational Health Service in the UKPCLPost-traumatic stress disorder Check ListPHQPatient Health Questionnaire

### Prevalence of PCS and most common symptoms

Across both timepoints, 1730 (33.6%) HCWs with a previous COVID-19 infection reported symptoms lasting ≥12 weeks, consistent with PCS (table 3). Of these, 385 also reported a formal diagnosis of LC or PCS (22.2% of those meeting criteria for PCS; 7.4% of all reporting a COVID-19 infection). The most common PCS symptom was fatigue, followed by difficulty concentrating, insomnia, anxiety/depression, shortness of breath and memory loss/confusion. See online supplemental table 5a,b for the prevalence of each symptom at 12 and 32 months, respectively. Additionally, 69.3% reported 2 or more PCS symptoms, and 7% reported a diabetes diagnosis following COVID-19 infection (online supplemental table 6).

**Table 3:** Prevalence of COVID-19 symptoms and their duration in the sample of HCWs who reported a previous COVID-19 infection in NHS CHECK (n=5248)

	No reported symptoms	Symptoms for <4 weeks (acute infection)	Symptoms for ≥4 weeks but <12 weeks (OSC)	Symptoms for ≥12 weeks (PCS)	
	n (%)	n (%)	n (%)	n (%)	(%), PCS only
Duration of any COVID-19 symptoms					
Experienced any symptom	287 (5.4)	2591 (49.2)	640 (11.8)	1730* (33.6)	–
Duration of each COVID-19 symptom					
Fatigue	710 (14.3)	3032 (57.2)	613 (11.0)	893 (17.5)	(51.4)
Difficulty concentrating	2832 (54.7)	1427 (26.6)	321 (6.1)	668 (12.6)	(36.7)
Insomnia	2879 (55.5)	1500 (27.9)	245 (4.9)	624 (11.7)	(35.2)
Anxiety or depression	3469 (65.2)	1023 (19.8)	209 (3.9)	547 (11.1)	(33.0)
Shortness of breath	2423 (45.8)	1963 (37.7)	351 (6.7)	511 (9.8)	(28.6)
Memory loss or confusion	3640 (69.7)	904 (17.3)	210 (3.7)	494 (9.3)	(27.0)
Loss or change in sense of taste/smell	2409 (46.0)	2106 (39.6)	318 (6.2)	415 (8.2)	(23.4)
Joint pains	2003 (38.4)	2636 (49.5)	203 (4.5)	406 (7.6)	(23.0)
Muscle or body aches	1090 (21.0)	3584 (67.5)	220 (4.3)	354 (7.2)	(21.7)
Tinnitus	4300 (81.3)	604 (12.6)	44 (0.8)	300 (5.3)	(15.5)
Headache	1151 (22.6)	3613 (67.5)	216 (4.6)	268 (5.3)	(15.5)
Chest pain or tightness	3097 (59.0)	1646 (31.6)	228 (4.3)	277 (5.1)	(15.3)
Heart palpitations	4005 (74.8)	872 (17.8)	130 (2.5)	241 (4.9)	(14.7)
Cough	1498 (28.9)	3130 (58.9)	374 (6.9)	246 (4.3)	(12.8)
Pins and needles or numbness	4337 (81.7)	614 (12.9)	70 (1.3)	227 (4.1)	(12.4)
Dizziness	3528 (67.0)	1355 (25.9)	154 (3.1)	211 (4.0)	(12.3)
Runny nose or nasal congestion	1915 (38.1)	2958 (54.8)	166 (3.1)	209 (4.0)	(11.4)
Sore throat	1893 (37.9)	3135 (58.0)	121 (2.3)	99 (1.8)	(5.3)
Stomach pain	4099 (77.3)	996 (19.8)	60 (1.1)	93 (1.8)	(5.3)
Sneezing	2665 (52.0)	2416 (44.9)	74 (1.4)	93 (1.7)	(5.0)
Skin rash	4494 (84.6)	621 (13.0)	46 (0.9)	87 (1.5)	(4.6)
Diarrhoea	3774 (71.4)	1350 (26.3)	56 (0.9)	68 (1.4)	(4.0)
Fever, chills or shivering	1459 (26.8)	3656 (70.3)	68 (1.6)	65 (1.3)	(4.0)
Nausea or vomiting	3834 (72.8)	1290 (25.0)	57 (0.9)	67 (1.3)	(3.9)

% = percentages weighted based on sex, age, ethnicity and job role of workforce in participating Trusts; %, PCS only = weighted percentages only including staff who met the definition of PCS.

COVID-19 symptoms are ordered by the prevalence of PCS symptoms in the sample.

* This includes 385 HCWs who reported a formal Long COVID/PCS diagnosis received from a medical professional.

HCWshealthcare workersnunweighted frequenciesOSCOngoing Symptomatic COVID-19PCSpost COVID-19 syndrome

### Risk factors for PCS

We found a very strong association between meeting the GHQ-12 cut-off score at baseline, indicating probable CMDs, and reporting PCS symptoms at follow-up (table 4). Having direct contact with COVID-19 patients also had a very strong association with reporting PCS symptoms, while having asthma/COPD or female sex had a strong association. Associations were also observed for age, as those aged between 51 and 60 were more likely to report PCS, and job role, where doctors were less likely to report PCS.

**Table 4:** Results from the multilevel logistic regression exploring baseline risk factors for reporting post COVID-19 syndrome at least 12 months later among the HCWs included in NHS CHECK (n=5248)

Baseline variables	Odds of reporting PCS	
	aOR	95% CI
Probable common mental disorders		
No (GHQ-12 score <4; Ref)	1	–
Yes (GHQ-12 score ≥4)	1.91***	(1.65, 2.23)
Sex		
Female (Ref)	1	–
Male	0.80*	(0.67, 0.94)
Age in years		
30 and younger (Ref)	1	–
31–40	1.50	(0.82, 2.75)
41–50	1.71	(0.94, 3.14)
51–60	1.81***	(1.32, 2.47)
61 and older	1.46	(0.92, 2.31)
Ethnicity		
White (Ref)	1	–
Black	0.73	(0.30, 1.75)
Asian	0.75	(0.39, 1.43)
Mixed/multiple ethnic group	0.88	(0.40, 1.91)
Other ethnic group	1.17	(0.49, 2.82)
Job role		
Nurse (Ref)	1	–
Doctor	0.64*	(0.42, 0.97)
Other clinical	0.75	(0.57, 1.01)
Non-clinical	0.78	(0.60, 1.01)
Relationship status		
Single/divorced (Ref)	1	–
Married/cohabitating	1.04	(0.83, 1.30)
Probable alcohol use disorder		
No (AUDIT-C score <8; Ref)	1	–
Yes (AUDIT-C score ≥8)	0.94	(0.68, 1.30)
Pre-existing respiratory illness		
No (Ref)	1	–
Reported asthma/COPD	1.54*	(1.13, 2.11)
Income		
NHS Band 5 or below (Ref)	1	–
NHS Band 6 or above	0.82	(0.61, 1.11)
Contact with COVID-19 patients		
No (Ref)	1	–
Yes	1.70***	(1.31, 2.21)
Perceived access to personal protective equipment		
Perceived inadequate access (Ref)	1	–
Perceived adequate access	0.59	(0.32, 1.09)
Non-applicable	0.71	(0.35, 1.42)
Confidence in infection control policies		
Inadequate confidence (Ref)	1	–
Adequate confidence	0.98	(0.83, 1.17)

aOR = adjusted OR, adjusted for all variables in the table, as well as burden on NHS when risk factor data were collected and Trust.

NHS Band 5 or below = annual income of £34 581 or lower; NHS Band 6 or above = annual income of £35 392 or higher (as of January 2024).

*p<0.05; **p≤0.01; ***p≤0.001.

COPDchronic obstructive pulmonary diseaseGHQGeneral Health QuestionnaireHCWshealthcare workersNHSNational Health Service in the UKPCSpost COVID-19 syndrome (symptoms lasting for 12 or more weeks following acute COVID-19 infection)

Scoring above the GHQ-12 cut-off score maintained a very strong association in sensitivity analyses using formal diagnosis of LC/PCS as the outcome (online supplemental table 7), the broad definition of LC as the outcome (symptoms for 4+weeks^1^ ; online supplemental table 8) and data collected at 12 and 32 months separately (online supplemental table 9a–c). Hospitalisation during the acute COVID-19 infection had a very strong association with reporting PCS symptoms at 12 months (online supplemental table 9b). Meeting the PHQ-9, GAD-7, BAT-12 and PCL-6 cut-off scores at baseline all had a very strong association with reporting PCS symptoms at follow-up (online supplemental table 10a–d). Differences were observed in the other risk factors. Having contact with COVID-19 patients was not a risk factor when including hospitalisation during the acute infection, asthma/COPD was not a risk factor using only 12-month data, and female sex was not a risk factor using formal LC/PCS diagnosis as the outcome or only 32-month data. Age and job role were risk factors for reporting PCS symptoms in most models, but the observed associations between levels of these variables varied. Due to this variability, we do not focus on these in our discussion. See the online supplemental materials for commentary on the MICE procedure.

## Discussion

Our finding that one-third of HCWs reported prolonged COVID-19 symptoms consistent with PCS is comparable to the results of the REal-time Assessment of Community Transmission (REACT) survey, where 37.7% of their representative UK community sample reported at least one COVID-19 symptom lasting ≥12 weeks.^30^ Despite this, only 7.4% of all HCWs in our study reported a formal diagnosis of LC/PCS. This is similar to the results of a meta-analysis which found that measuring PCS by self-reported symptoms, as in this study, estimated significantly higher prevalences than healthcare records,^9^ while another study found that only ~9% of HCWs who reported COVID-19 symptoms for ≥12 weeks also reported a formal diagnosis.^31^ We theroise it may be that self-reported measures overestimate prevalence and may not alone be specific enough to correctly identify those impacted by PCS. For example, given that fatigue was our most prevalent symptom and HCWs often work shifts, it could be unsurprising that a high proportion met the definition of PCS. We think that it could also be that those experiencing symptoms do not attempt to obtain a diagnosis, believing that nothing can be done to help them, their symptoms are mild and do not require care, or they will recover in time. Alternatively, it may be that those who do request a diagnosis are not given one.

Our findings highlight the complexity of PCS symptoms. The most common PCS symptom was fatigue, and most experienced cognitive symptoms (66.2%), such as difficulty concentrating and memory loss. The majority reported multiple PCS symptoms (69.3%), and some attributed the development of new conditions to their COVID-19 infection. In particular, 7% reported a diagnosis of type 1 or 2 diabetes following COVID-19, fitting with previous work which found a link between COVID-19 and increased risk of diabetes.^32^

Our analysis also found that screening positive for mental disorders, direct contact with COVID-19 patients, having a pre-existing respiratory condition, female sex and older age at baseline were risk factors for reporting PCS at follow-up. Additionally, sensitivity analyses of the 12-month data indicated that hospitalisation during the acute infection was a risk factor. Without a known pathogenesis of PCS, it is difficult to speculate why these were risk factors, though studies with similar results have proposed explanations.^1133^,^37^ These have speculated that symptoms of CMDs may be a risk factor due to potential physical manifestation of psychological distress,^11 36^ while female sex and older age may be associated with PCS due to overlap between PCS and menopausal symptoms.^37^ Contact with COVID-19 patients may be a risk factor as these HCWs may have experienced multiple COVID-19 infections, a known risk factor.^10^ It may also be that HCWs who worked with COVID-19 patients had greater knowledge of the condition than those who did not and were better able to identify their symptoms. While we do not identify any individual in this study as having ME/CFS, we note the similarity between not just symptoms, as many have pointed out, but also at least five risk factors for PCS and ME/CFS (infection, asthma, a history of CMDs, female sex and older age) which should not be ignored and similarities between the conditions may require further exploration.^5^

The strongest observed statistical association among risk factors for PCS was reporting symptoms of CMDs at baseline, which also included specific measures for depression, GAD, PTSD and burnout. The majority of HCWs in our sample had not tested for COVID-19 or had received a negative test result prior to reporting baseline data, indicating that for most, symptoms of CMDs predated COVID-19. Despite emerging evidence that CMDs are risk factors for PCS, research examining this remains sparse.^10 11 33 36^ This may raise potentially controversial questions regarding the implications of significant findings. For example, Burton *et al* admitted to excluding measures of mood or cognitive features when looking for risk factors for PCS as ‘patient input into the design of the study saw this as potentially implying psychological causation of symptoms’.^38^ While acknowledging this challenge, we argue that it is vital to examine the relationship between CMDs and PCS, and failure to confirm or refute this will weaken our understanding of the condition.

The primary strength of this study was its longitudinal nature, which allowed us to examine risk factors for PCS that were measured at least 1 year before staff reported COVID-19 data. Additionally, using data provided by each Trust, our sample was weighted by sex, age, ethnicity and job role to better represent the population from which they were drawn. Finally, our PPIE group helped to ensure that this study addressed issues relevant to HCWs with experience of PCS.

Our results must be considered within some limitations. First, the symptoms used to identify PCS are not condition specific and may be prevalent regardless of previous COVID-19 infections. This may have contributed to the difference in the number of HCWs who reported symptoms consistent with PCS (33.6%) and who reported a formal diagnosis of LC or PCS (7.4%). As we only asked those who reported a previous COVID-19 infection at 12 and 32 months to describe what symptoms they experienced, we cannot approximate the general prevalence of each symptom in our sample. Two solutions may have been to ask the symptom questions to all participants either at baseline or 12-month and 32-month follow-up. Future NHS CHECK data collection waves will ask symptom questions to all participants. There may also be recall bias in symptom prevalence, as participants were asked to self-report how long they experienced each symptom for, perhaps after they had fully recovered.

Relatedly, the lack of a consensus definition of LC is a problem within this field.^39^ We included symptoms based on guidance from NICE at the time of data collection,^1^ and note some symptoms in other studies were not measured here. Several other definitions exist, such as post COVID-19 condition from the WHO.^2^ Differing definitions, symptoms and timeframes used to denote LC make it difficult to compare results across studies, and differences in observed risk factors may exist based on how the condition is defined. We reflect on this in greater detail in a recent review.^40^ We also did not examine PCS-symptom severity, and it may be that those most impacted did not participate. Additionally, we intended to examine if hospitalisation during the acute infection was a risk factor for reporting PCS; however, this was erroneously omitted from the 32-month survey. Sensitivity analysis indicated that hospitalisation was a risk factor at 12 months. Finally, certain variables which have been found to be risk factors for PCS were not collected by NHS CHECK, such as number of acute COVID-19 infections, vaccination against COVID-19, body mass index and comorbidities, such as diabetes.^10^ These omissions may have contributed to residual confounding in our analyses.

## Conclusions

The difference between the number of HCWs reporting symptoms consistent with PCS and those reporting a formal diagnosis of LC or PCS is striking. We also confirm previous reports that baseline symptoms of CMDs increase the risk of PCS, while bearing in mind that the majority of studies exploring PCS lack this risk factor. The cause, or more likely causes, of PCS remain unclear and may do so for some time. Until further research leads to more specific treatments, the need to support and provide rehabilitation for those with PCS remains.

## supplementary material

10.1136/oemed-2024-109621 online supplemental file 1

10.1136/oemed-2024-109621 online supplemental file 2

10.1136/oemed-2024-109621 online supplemental file 3

## Data Availability

Data are available upon reasonable request.
