# Supplementary figures and images for: Post COVID-19 syndrome among 5248 healthcare workers in England: longitudinal findings from NHS CHECK
Source: Occup Environ Med. 2024 Oct 2;81(9):e109621. doi: 10.1136/oemed-2024-109621 (PMC11503206; doi:10.1136/oemed-2024-109621)

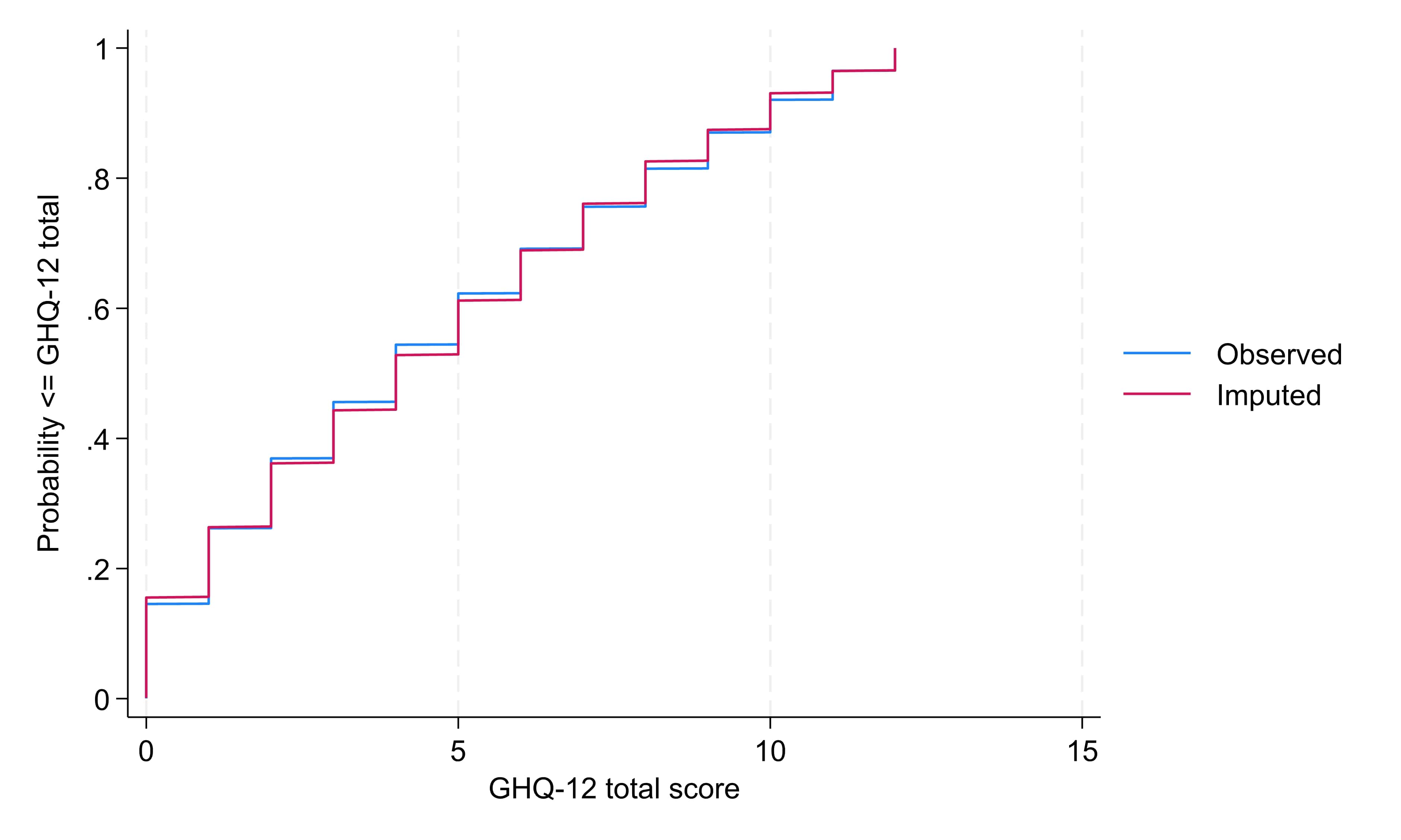

Supplement: online supplemental file 1 [file oemed-81-9-s001.jpg]

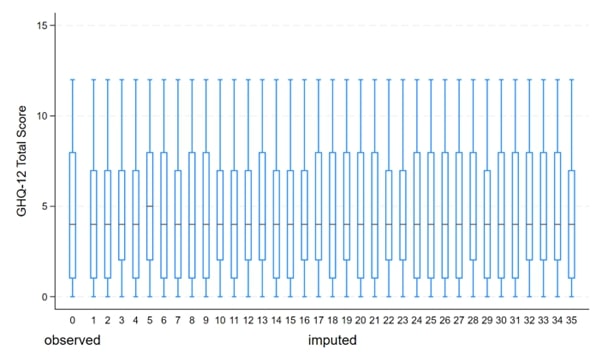

Supplement: online supplemental file 2 [file oemed-81-9-s002.jpg]
