# Supplementary material for: Post COVID-19 syndrome among 5248 healthcare workers in England: longitudinal findings from NHS CHECK
Source: Occup Environ Med. 2024 Oct 2;81(9):e109621. doi: 10.1136/oemed-2024-109621 (PMC11503206; doi:10.1136/oemed-2024-109621)
Supplement: online supplemental file 3 [file oemed-81-9-s003.docx]

**Post Covid-19 Syndrome among 5,248 healthcare workers in England: Longitudinal findings from NHS CHECK.**

**Supplementary Materials**

**Section A**

**Detailed Description of the Multiple Imputation using Chained Equations (MICE) Procedure**

To account for missing data in our risk factor variables and maintain power in our calculations, Multiple Imputation using Chained Equations (MICE) was used.

To examine if the data were missing at random, and thus appropriate for MICE, we considered the three distinct mechanisms responsible for missing values. Firstly, the 4,603 HCWs that completed the baseline survey were exposed to all variables (excluding the secondary mental disorder variables), and thus missingness is based on individual non-response to the questions. Most have complete data for all of the variables included in the short version of the baseline survey (*n* = 3,062, 66.5%; see Supplemental Table 1a). Of the remaining 1,541 HCWs with missing data, 978 (63.5%) only had missing values for two variables: 546 only had missing data for contact with Covid-19 patients, 376 only had missing data for income, and a further 56 only had missing values for both. For contact with Covid-19 patients, most of the missing values were not truly missing, as the original question gave participants the option to respond “unsure”, which was recoded as missing for analysis. For income, we believe that people may have chosen to skip this question given cultural views about discussing income, hence the 11.4% missing data. Secondly, of the 4,603 HCWs that completed the baseline survey, only 3,155 completed the longer version, and thus saw the secondary mental disorder variables. To investigate whether choosing to complete the longer baseline survey or the baseline short only could be considered a random process, we compared the distribution of the other risk factor data between these groups. While chi-square tests revealed differences in the distribution of some risk factor variables between those who completed the longer baseline survey or the baseline short version only (see Supplemental Table 1b), we concluded that these deviations were unlikely to indicate a systematic reason why some HCWs did not complete the longer baseline survey version. Thirdly, some of the risk factor variables were not asked on the 12 month survey, meaning that the 645 HCWs who comprised the replenishment sample had missing data for all. These included all mental disorder variables, pre-existing respiratory illness, contact with Covid-19 patients, perceived access to PPE, and confidence in infection control policies. As we recruited the replenishment sample from HCWs in the same 18 NHS Trusts as the baseline survey, we believe that it may be completely random whether staff joined at the original baseline survey or at the 12 month follow-up. Considering each mechanism, we believe that MICE is suitable for this study.

Our next step was to build the imputation model. All the risk factor variables, along with the outcome variables, were included in the imputation model to reduce bias. Auxiliary variables included in the imputation model comprised: feelings of managerial, collegial, and familial support, contract permanency, and intentions to leave their job when they reported their Covid-19 infection and symptoms. These auxiliary variables are to be included in a different analysis. Where we had access to longitudinal measurements for the risk factors (i.e. for the mental disorder variables), participant’s earliest follow-up score was included as an auxiliary variable. For the GHQ-12, PHQ-9, GAD-7, PCL-6 and AUDIT-C follow-up measurements, more than 99% of participants in this study had a completed score for each. The exception was BAT-12, for which 13.1% did not have a follow-up score.

As we did not seek to impute any values for our outcome variable (reporting symptoms of PCS), all participants that had not reported a previous Covid-19 infection at 12 or 32-month follow-up were excluded. Where appropriate, we included the continuous version of the risk factor variables in the imputation model over the categorical versions (i.e. age and total scores on each mental disorder scale, rather than the ordinal age categories and binary mental disorder cutoff scores). The continuous variables were then recoded into categories, that were included in the subsequent analyses. As these continuous variables were not normally distributed, predictive mean matching was used to draw imputations for the missing values, where *knn*=10. We chose *knn* = 10 following a report from Morris et al. that larger values should be preferred (1). Additionally, where variables originated from a multi-item scale (i.e. the mental disorder variables), we opted to impute missing values for the scale total rather than the individual items (2). This decision was made as the majority of participants who had missing data for the scale totals did not answer any of the questions. Following a suggestion from White et al. that there should be at least as many imputed datasets as the percentage of participants with missing data (3), we generated 70 completed datasets given that approximately 68.1% (*n* = 3,572) of the sample had missing data on at least one of the risk factor variables.

As a post hoc assessment of the imputation model, all imputed scores for each variable were checked to ensure that they fell within the expected ranges. Following guidance from Nyguen et al. (4), we also visually examined the imputed values against the observed scores for each variable. See Supplemental Figures 1 and 2 for examples of this for our primary mental disorder variable, GHQ-12. Supplemental Figure 1 is a cumulative distribution plot comparing observed (blue line) and imputed (red line) GHQ-12 total scores for a single imputed dataset. Supplemental Figure 2 gives boxplots for the observed GHQ-12 total scores (labelled 0) and the first 35 imputed datasets (50%; labelled 1 – 35). In summary, we found no significant visual deviations in the imputed data, suggesting that the model had been successful in imputing realistic values. We also examined the descriptive statistics between the observed and imputed values for risk factors that were found to have a strong or very strong association with reporting PCS symptoms and had ≥10% missing data (see Table 4 and Supplemental Tables 10a-d). Though there were deviations between some of the statistics for the observed and imputed data, we believed them to be appropriate and evidence of a plausible imputation.

In addition to checking the imputed values against the observed values, we also conducted some sensitivity analyses to examine the MICE procedure. Firstly, as we chose to impute missing values for all variables with any degree of missingness in the analysis presented in the main article, we conducted a second MICE procedure only imputing values for variables that had ≥5% missingness. The same parameters as above were used for this second imputation model and a multi-level logistic regression was conducted using data from the newly imputed datasets to estimate risk factors for reporting PCS symptoms (Supplemental Table 4a). As in our main model (Table 4), we observed a very strong association between GHQ-12 cutoff score, age, and contact with Covid-19 patients and odds of reporting PCS symptoms, and a strong association between sex and pre-existing respiratory illness and reporting PCS symptoms. Secondly, we also conducted a complete cases analysis (Supplemental Table 4b). This model included 1,987 HCWs (37.8% of the sample) who had complete data for each of the risk factor variables included in our analyses. Again, compared to our main model (Table 4), we observed a very strong association between GHQ-12 cutoff score and contact with Covid-19 patients and odds of reporting PCS symptoms, and a strong association between age and pre-existing respiratory illness and reporting PCS symptoms. Sex, however, was no longer a risk factor for reporting PCS symptoms when considering complete cases only. Given the exclusion of over 3,000 participants in this model and the fact that sex had a low level of missing data, the complete cases analysis highlights a bias, signalling a benefit to including MICE in this study. Finally, while the utilisation of MICE in this study helped to maintain statistical power in our analyses and were deemed to be appropriate, it may be possible that there was bias in our calculations if the data were missing not at random. Acknowledging this, and due to multi-collinearity, we chose to include the mental disorder variables with the highest proportion of missing data (e.g. PHQ-9, GAD-7, BAT-12, and PCL-6) in secondary analyses only. Despite this potential limitation and based on our preliminary investigations, it is reasonable that we may assume the data is missing at random.

1. Morris TP, White IR, Royston P. Tuning multiple imputation by predictive mean matching and local residual draws. BMC Med Res Methodol. 2014 Dec;14(1):75.

2. White IR, Royston P, Wood AM. Multiple imputation using chained equations: Issues and guidance for practice. Statist Med. 2011 Feb 20;30(4):377–99.

3. Nguyen CD, Carlin JB, Lee KJ. Model checking in multiple imputation: an overview and case study. Emerg Themes Epidemiol. 2017 Dec;14(1):8.

**Supplemental Table 1a**. The number and percentage of complete and missing responses for each of the risk factor variables included in the short version of the baseline NHS CHECK survey, which was completed by 4,603 HCWs.

| **Variable** | **Complete** | **%** | **Missing** | **%** |
| --- | --- | --- | --- | --- |
| Age | 4,402 | 95.6 | 201 | 4.4 |
| Sex | 4,565 | 99.2 | 38 | 0.8 |
| Relationship status | 4,576 | 99.4 | 27 | 0.6 |
| Ethnicity | 4,579 | 99.5 | 24 | 0.5 |
| Job role | 4,580 | 99.5 | 23 | 0.5 |
| Income | 4,080 | 88.6 | 523 | 11.4 |
| Contact with Covid-19 patients | 3,851 | 83.7 | 752 | 16.3 |
| Confidence in infection control | 4,480 | 97.3 | 123 | 2.7 |
| Access to PPE | 4,481 | 97.3 | 122 | 2.7 |
| Pre-existing respiratory illness | - | - | - | - |
| GHQ-12 | 4,341 | 94.3 | 262 | 5.7 |
| All variables | 3,062 | 66.5 | 1,541 | 33.5 |
| HCWs = healthcare workers; NHS = National Health Service; PPE = personal protective equipment; GHQ = general health questionnaire.  Note. Variables are presented in the order in which they appeared in the baseline survey. Note that there are no missing data for pre-existing respiratory illness as there was not a “no” option for this question. | | | | |

**Supplemental Table 1b.** Results of chi-square analyses comparing the baseline risk factor information of HCWs who chose to complete the longer baseline survey version (*n* = 12,596) and those who only chose to complete the short baseline survey (*n* = 9,958).

|  |  | **Baseline Longer survey** | | **Baseline Short survey only** | | ***p* value** |
| --- | --- | --- | --- | --- | --- | --- |
| **Characteristic** | **Categories** | ***n*** | **%** | ***n*** | **%** |  |
| Probable CMDs | Yes (GHQ-12 score ≥4) | 6,829 | 44.1 | 4,141 | 51.7 | <0.001 |
|  | No (GHQ-12 score <4; Ref) | 5,377 | 55.9 | 4,434 | 48.3 |  |
|  | Missing | 390 (3.1) | | 1,383 (13.9) | |  |
| Sex | Female | 10,412 | 83.1 | 7,712 | 78.9 | <0.001 |
|  | Male | 2,120 | 16.9 | 2,057 | 21.1 |  |
|  | Missing data | 64 (0.5) | | 189 (1.9) | |  |
| Age (years) | ≤30 | 2,213 | 18.3 | 2,084 | 22.2 | <0.001 |
|  | 31-40 | 2,720 | 22.5 | 2,223 | 23.6 |  |
|  | 41-50 | 3,174 | 26.3 | 2,464 | 26.2 |  |
|  | 51-60 | 3,167 | 26.2 | 2,120 | 22.5 |  |
|  | ≥61 | 815 | 6.7 | 517 | 5.5 |  |
|  | Missing data | 507 (4.0) | | 550 (5.5) | |  |
| Ethnicity | White | 11,254 | 89.6 | 7,916 | 80.7 | <0.001 |
|  | Black | 338 | 2.7 | 635 | 6.5 |  |
|  | Asian | 603 | 4.8 | 870 | 8.9 |  |
|  | Mixed/Multiple ethnic group | 281 | 2.2 | 265 | 2.7 |  |
|  | Other ethnic group | 88 | 0.7 | 121 | 1.2 |  |
|  | Missing | 32 (0.3) | | 151 (1.5) | |  |
| Job role | Nurse | 3,391 | 27.0 | 2,387 | 24.3 | <0.001 |
|  | Doctor | 830 | 6.6 | 808 | 8.2 |  |
|  | Other clinical | 3,659 | 29.1 | 3,112 | 31.7 |  |
|  | Non-clinical | 4,700 | 37.3 | 3,506 | 35.8 |  |
|  | Missing data | 16 (0.1) | | 145 (1.5) | |  |
| Relationship status | In a relationship | 9,395 | 25.1 | 7,131 | 27.1 | 0.001 |
|  | Single | 3,152 | 74.9 | 2,651 | 72.9 |  |
|  | Missing data | 49 (0.4) | | 176 (1.8) | |  |
| Pre-existing respiratory illness | None | 11,855 | 94.1 | 9,483 | 95.2 | <0.001 |
|  | Reported asthma/COPD | 741 | 5.9 | 475 | 4.8 |  |
| Income | NHS Band 5 or below | 4,169 | 37.1 | 2,963 | 36.9 | 0.827 |
|  | NHS Band 6 or above | 7,081 | 62.9 | 5,066 | 63.1 |  |
|  | Missing | 1,346 (10.7) | | 1,929 (19.4) | |  |
| Contact with Covid-19 patients | No contact | 4,597 | 43.2 | 3,603 | 45.8 | <0.001 |
|  | Contact | 6,048 | 56.8 | 4,261 | 54.2 |  |
|  | Missing | 1,951 (15.5) | | 2,094 (21.0) | |  |
| Perceived access to PPE | Perceived inadequate access | 972 | 7.8 | 736 | 8.0 | 0.275 |
|  | Perceived adequate access | 9,828 | 78.8 | 7,302 | 79.3 |  |
|  | Non-applicable | 1,672 | 13.4 | 1,167 | 12.7 |  |
|  | Missing | 123 (1.0) | | 753 (7.6) | |  |
| Confidence in infection control policies | Inadequate confidence | 4,782 | 38.4 | 3,429 | 37.4 | 0.122 |
|  | Adequate confidence | 7,664 | 61.6 | 5,742 | 62.5 |  |
|  | Missing | 150 (1.2) | | 787 (7.9) | |  |
| Covid-19 testing status at time of baseline survey response * | Tested positive for Covid-19 | 707 | 5.7 | 547 | 6.0 | 0.543 |
|  | Tested negative for Covid-19 | 4,251 | 34.0 | 3,111 | 34.1 |  |
|  | Tested, awaiting result | 144 | 1.1 | 93 | 1.0 |  |
|  | Tested, inconclusive result | 88 | 0.7 | 75 | 0.8 |  |
|  | Never tested | 7,316 | 58.5 | 5,296 | 58.1 |  |
|  | Missing | 90 (0.7) | | 836 (8.4) | |  |
| *p* value represents the results of chi-square tests comparing the distribution of HCWs for each of the variables based on whether they completed the longer version of the baseline survey or only completed the short version. The % column presents the distribution of each characteristic for observed data only. The missing row gives the percentage for each variable that had missing data.  CMDs = common mental disorder; HCWs = healthcare workers; GHQ = general health questionnaire; COPD = Chronic Obstructive Pulmonary Disease; NHS = National Health Service in the United Kingdom; NHS Band 5 or below = Annual income of £34,581 or lower; NHS Band 6 or above = Annual income of £35,392 or higher (As of January 2024); PPE = personal protective equipment.  Table does not include the 1,583 HCWs that began from the replenishment sample. | | | | | | |

[Insert Supplemental Figure 1 here]

[Insert Supplemental Figure 2 here]

**Supplemental Table 2.** Summary statistics for the observed and imputed data for the incomplete variables that were found to be risk factors for reporting Post Covid-19 Syndrome in the multi-level regression model and had ≥10% missing data before Multiple Imputation via Chained Equations.

| **Risk Factors** |  | **Observed Data** | | | | | **Imputed Data** | | | | |
| --- | --- | --- | --- | --- | --- | --- | --- | --- | --- | --- | --- |
|  | **Variable** | ***n*** | **Mean** | **SD** | **Min-Max** | **%*** | ***n*** | **Mean** | **SD** | **Min-Max** | **%*** |
| **Probable common mental disorders** | GHQ-12 total score | 4,341 | 4.66 | 3.63 | 0 – 12 | 54.87 | 907 | 4.63 | 4.86 | 0 – 12 | 54.57 |
| **Probable depression** | PHQ-9 total score | 3,030 | 7.36 | 5.76 | 0 – 27 | 29.37 | 2,218 | 7.11 | 6.82 | 0 – 27 | 27.34 |
| **Probable generalised anxiety disorder** | GAD-7 total score | 3,045 | 6.32 | 5.08 | 0 – 21 | 22.10 | 2,203 | 6.09 | 5.93 | 0 – 21 | 20.03 |
| **Probable burnout** | BAT-12 total score | 2,998 | 2.28 | 0.69 | 1 – 5 | 16.21 | 2,250 | 2.24 | 0.82 | 1 – 5 | 14.69 |
| **Probable post-traumatic stress disorder** | PCL-6 total score | 3,033 | 11.05 | 4.50 | 6 – 30 | 24.07 | 2,215 | 10.82 | 5.73 | 6 – 30 | 22.02 |
| **Probable alcohol use disorder** | AUDIT-C total score | 2,871 | 3.70 | 3.81 | 0 – 31 | 11.74 | 2,377 | 3.76 | 5.00 | 0 – 31 | 12.42 |
| **Pre-existing respiratory disorder** | Reported asthma/COPD | 4,603 | – | – | – | 6.69 | 645 | – | – | – | 7.32 |
| **Contact with Covid-19 patients** | Yes | 3,851 | – | – | – | 60.37 | 1,397 | – | – | – | 59.66 |
| The summary statistics of the imputed data were calculated using pooled data over 70 imputations. Post Covid-19 Syndrome occurs when symptoms of Covid-19 last for 12 or more weeks following acute Covid-19 infection. COPD = chronic obstructive pulmonary disease; HCWs = healthcare workers.  *For the mental disorder scales, this refers to the percentage of HCWs meeting the cut-off score on each: GHQ-12 total score ≥4; PHQ-9 total score ≥10; GAD-7 total score ≥10; BAT-12 total score ≥2.96; PCL-6 total score ≥14; AUDIT-C total score ≥8. For pre-existing respiratory disorder and contact with Covid-19 patients, this refers to the percentage of HCWs that reported asthma or COPD or the number of HCWs that had contact with Covid-19 patients. | | | | | | | | | | | |

**Supplemental Table 3a.** Results from the Multi-Level Logistic Regression exploring baseline risk factors for reporting Post Covid-19 Syndrome at least 12 months later among the HCWs included in NHS CHECK, when only imputing values for variables missing more than 5% of the responses (n = 5,103).

| **Variable** |  | **Odds of Reporting PCS** | |
| --- | --- | --- | --- |
|  | **Categories** | **aOR** | **95% CI** |
| Probable common mental disorders | No (GHQ-12 score <4; Ref) | 1 | – |
|  | Yes (GHQ-12 score ≥4) | 1.91*** | [1.61, 2.27] |
| Sex | Female (Ref) | 1 | – |
|  | Male | 0.78** | [0.66, 0.93] |
| Age in years* | 30 and younger (Ref) | 1 | – |
|  | 31–40 | 1.41 | [0.83, 2.41] |
|  | 41–50 | 1.62 | [0.96, 2.74] |
|  | 51–60 | 1.67*** | [1.32, 2.12] |
|  | 61 and older | 1.34 | [0.80, 2.25] |
| Ethnicity | White (Ref) | 1 | – |
|  | Black | 0.74 | [0.29, 1.90] |
|  | Asian | 0.74 | [0.37, 1.48] |
|  | Mixed/Multiple ethnic group | 0.87 | [0.40, 1.88] |
|  | Other ethnic group | 1.27 | [0.53, 3.05] |
| Job role | Nurse (Ref) | 1 | – |
|  | Doctor | 0.64* | [0.42, 0.95] |
|  | Other clinical | 0.74 | [0.53, 1.03] |
|  | Non-clinical | 0.77 | [0.58, 1.02] |
| Relationship status | Single/Divorced (Ref) | 1 | – |
|  | Married/Cohabitating | 1.07 | [0.86, 1.32] |
| Probable alcohol use disorder* | No (AUDIT-C score <8; Ref) | 1 | – |
|  | Yes (AUDIT-C score ≥8) | 0.92 | [0.64, 1.32] |
| Pre-existing respiratory illness* | No (Ref) | 1 | – |
|  | Reported Asthma/COPD | 1.78* | [1.16, 2.75] |
| Income | NHS Band 5 or below (Ref) | 1 | – |
|  | NHS Band 6 or above | 0.83 | [0.60, 1.15] |
| Contact with Covid-19 patients* | No (Ref) | 1 | – |
|  | Yes | 1.73*** | [1.33, 2.24] |
| Perceived access to PPE* | Perceived inadequate access (Ref) | 1 | – |
|  | Perceived adequate access | 0.60 | [0.34, 1.05] |
|  | Non-applicable | 0.70 | [0.35, 1.38] |
| Confidence in infection control policies* | Inadequate confidence (Ref) | 1 | – |
|  | Adequate confidence | 0.97 | [0.81, 1.16] |
| HCWs = Healthcare workers; PCS = Post Covid-19 Syndrome (symptoms lasting for 12 or more weeks following acute Covid-19 infection); aOR = Adjusted Odds Ratio, adjusted for all variables in the table, as well as burden on NHS when risk factor data were collected and Trust; 95% CI = 95% Confidence Intervals; COPD = Chronic Obstructive Pulmonary Disease; NHS = National Health Service in the United Kingdom; NHS Band 5 or below = Annual income of £34,581 or lower; NHS Band 6 or above = Annual income of £35,392 or higher (As of January 2024). * Indicating variables that had more than 5% missing data and were imputed. * *p* < 0.05; ** *p* ≤ 0.01; *** *p* ≤ 0.001. | | | |

**Supplemental Table 3b.** Results from the Multi-Level Logistic Regression exploring baseline risk factors for reporting Post Covid-19 Syndrome at least 12 months later among the HCWs included in NHS CHECK, using only data from participants who fully reported each of the included risk factor variables (*n* = 1,987).

| **Variable** |  | **Odds of Reporting PCS** | |
| --- | --- | --- | --- |
|  | **Categories** | **aOR** | **95% CI** |
| Probable common mental disorders | No (GHQ-12 score <4; Ref) | 1 | – |
|  | Yes (GHQ-12 score ≥4) | 2.35*** | [1.84, 3.00] |
| Sex | Female (Ref) | 1 | – |
|  | Male | 0.86 | [0.61, 1.20] |
| Age in years | 30 and younger (Ref) | 1 | – |
|  | 31–40 | 1.34 | [0.78, 2.30] |
|  | 41–50 | 1.55* | [1.07, 2.24] |
|  | 51–60 | 1.09 | [0.77, 1.53] |
|  | 61 and older | 1.37 | [0.45, 4.18] |
| Ethnicity | White (Ref) | 1 | – |
|  | Black | 1.16 | [0.30, 4.46] |
|  | Asian | 1.27 | [0.47, 3.44] |
|  | Mixed/Multiple ethnic group | 0.53 | [0.09, 3.00] |
|  | Other ethnic group | 0.84 | [0.16, 4.51] |
| Job role | Nurse (Ref) | 1 | – |
|  | Doctor | Empty | – |
|  | Other clinical | 0.79 | [0.60, 1.04] |
|  | Non-clinical | 0.81* | [0.66, 0.99] |
| Relationship status | Single/Divorced (Ref) | 1 | – |
|  | Married/Cohabitating | 0.92 | [0.60, 1.41] |
| Probable alcohol use disorder | No (AUDIT-C score <8; Ref) | 1 | – |
|  | Yes (AUDIT-C score ≥8) | 0.84 | [0.62, 1.14] |
| Pre-existing respiratory illness | No (Ref) | 1 | – |
|  | Reported Asthma/COPD | 2.32* | [1.16, 4.64] |
| Income | NHS Band 5 or below (Ref) | 1 | – |
|  | NHS Band 6 or above | 0.81 | [0.48, 1.35] |
| Contact with Covid-19 patients | No (Ref) | 1 | – |
|  | Yes | 1.79*** | [1.38, 2.31] |
| Perceived access to PPE | Perceived inadequate access (Ref) | 1 | – |
|  | Perceived adequate access | 0.51* | [0.30, 0.89] |
|  | Non-applicable | 0.49 | [0.21, 1.16] |
| Confidence in infection control policies | Inadequate confidence (Ref) | 1 | – |
|  | Adequate confidence | 0.99 | [0.77, 1.26] |
| HCWs = Healthcare workers; PCS = Post Covid-19 Syndrome (symptoms lasting for 12 or more weeks following acute Covid-19 infection); aOR = Adjusted Odds Ratio, adjusted for all variables in the table, as well as burden on NHS when risk factor data were collected and Trust; 95% CI = 95% Confidence Intervals; COPD = Chronic Obstructive Pulmonary Disease; NHS = National Health Service in the United Kingdom; NHS Band 5 or below = Annual income of £34,581 or lower; NHS Band 6 or above = Annual income of £35,392 or higher (As of January 2024). * *p* < 0.05; *** *p* ≤ 0.001. | | | |

**Section B**

**Additional Information on the Risk Factor and Outcome Variables**

**Supplemental Table 4a.** Baseline demographic information of the healthcare workers in NHS CHECK, given in terms of the total NHS CHECK cohort and for those who had a previous Covid-19 infection and who reported Post Covid-19 Syndrome for the 12 and 32 month follow-up surveys separately.

|  | | **Total NHS CHECK sample** | **12 Month Follow-Up Survey** | | **32 Month Follow-Up Survey** | |
| --- | --- | --- | --- | --- | --- | --- |
|  |  |  | **Staff with a Previous Covid-19 Infection** | **Staff with PCS** | **Staff with a Previous Covid-19 Infection** | **Staff with PCS** |
| **Demographic Information** | | ***n* = 24,137 (%)** | ***n* = 2,659 (%)** | ***n* = 1,040 (%)** | ***n* = 3,070 (%)** | ***n* = 816 (%)** |
| Sex | Female (Ref) | 19,381 (74.1) | 2,130 (74.9) | 878 (80.2) | 2,482 (73.9) | 679 (76.6) |
|  | Male | 4,491 (24.9) | 507 (24.3) | 156 (19.4) | 565 (25.3) | 130 (22.7) |
|  | Missing | 265 (1.0) | 22 (0.8) | 6 (0.4) | 23 (0.8) | 7 (0.7) |
| Age in years | 30 and younger (Ref) | 4,681 (21.3) | 463 (19.4) | 152 (15.6) | 339 (11.6) | 76 (9.1) |
|  | 31–40 | 5,247 (25.0) | 519 (22.6) | 181 (20.1) | 582 (23.1) | 147 (21.8) |
|  | 41–50 | 6,000 (22.0) | 694 (22.4) | 304 (25.3) | 906 (26.4) | 235 (25.6) |
|  | 51–60 | 5,691 (20.7) | 744 (25.9) | 309 (28.5) | 913 (26.9) | 265 (31.1) |
|  | 61 and older | 1,439 (6.5) | 135 (5.8) | 50 (5.2) | 212 (8.4) | 59 (8.4) |
|  | Missing | 1,079 (4.5) | 104 (3.9) | 44 (5.3) | 118 (3.6) | 34 (4.0) |
| Ethnicity | White (Ref) | 20,507 (75.9) | 2,288 (78.9) | 912 (81.7) | 2,814 (84.6) | 756 (86.5) |
|  | Black | 1,045 (8.0) | 107 (7.3) | 31 (5.4) | 57 (4.6) | 10 (4.0) |
|  | Asian | 1,572 (12.1) | 159 (10.0) | 58 (10.1) | 101 (7.5) | 17 (5.6) |
|  | Mixed/Multiple ethnic group | 593 (1.1) | 67 (1.2) | 26 (1.2) | 69 (1.3) | 22 (1.1) |
|  | Other ethnic group | 224 (2.2) | 21 (2.1) | 8 (1.3) | 16 (1.6) | 6 (2.2) |
|  | Missing | 196 (0.7) | 17 (0.5) | 5 (0.3) | 13 (0.4) | 5 (0.6) |
| Relationship status | Single/Divorced (Ref) | 6,304 (26.9) | 641 (23.4) | 246 (23.3) | 716 (24.5) | 189 (24.8) |
|  | Married/Cohabitating | 17,592 (72.2) | 2,002 (76.1) | 789 (76.4) | 2,335 (25) | 620 (74.4) |
|  | Missing | 241 (0.9) | 16 (0.5) | 5 (0.3) | 19 (0.5) | 7 (0.8) |
| n = unweighted frequencies (with weighted percentages); PCS = Post Covid-19 Syndrome (symptoms lasting for 12 weeks following acute Covid-19 infection); NHS = National Health Service in the United Kingdom | | | | | | |

**Supplemental Table 4b.** Baseline occupational information of the healthcare workers in NHS CHECK, given in terms of the total NHS CHECK cohort and for those who had a previous Covid-19 infection and who reported Post Covid-19 Syndrome for the 12 and 32 month follow-up surveys separately.

|  | | **Total NHS CHECK sample** | **12 Month Follow-Up Survey** | | **32 Month Follow-Up Survey** | |
| --- | --- | --- | --- | --- | --- | --- |
|  |  |  | **Staff with a Previous Covid-19 Infection** | **Staff with PCS** | **Staff with a Previous Covid-19 Infection** | **Staff with PCS** |
| **Occupational Information** | | ***n* = 24,137 (%)** | ***n* = 2,659 (%)** | ***n* = 1,040 (%)** | ***n* = 3,070 (%)** | ***n* = 816 (%)** |
| Job role | Nurse | 6,127 (29.7) | 761 (32.5) | 333 (36.5) | 754 (28.8) | 228 (32.2) |
|  | Doctor | 1,727 (9.8) | 200 (8.9) | 52 (5.9) | 176 (7.8) | 29 (4.6) |
|  | Other clinical | 7,338 (32.1) | 855 (33.9) | 345 (34.5) | 895 (31.8) | 224 (30.6) |
|  | Non-clinical | 8,744 (27.7) | 819 (24.1) | 302 (22.5) | 1,225 (30.9) | 331 (32.0) |
|  | Missing | 201 (0.7) | 24 (0.6) | 8 (0.6) | 20 (0.7) | 4 (0.6) |
| Income | NHS Band 5 or below | 7,599 (27.7) | 861 (28.9) | 345 (29.9) | 992 (24.7) | 270 (27.7) |
|  | NHS Band 6 or above | 12,570 (54) | 1,350 (54.0) | 557 (56.5) | 1,729 (60.2) | 442 (59.4) |
|  | Missing | 3,968 (18.3) | 448 (17.1) | 138 (13.6) | 419 (15.1) | 104 (12.9) |
| Contact with Covid-19 patients | No contact | 8,200 (27.1) | 545 (15.9) | 152 (12.6) | 1,077 (27.0) | 225 (22.3) |
|  | Contact | 10,309 (51.7) | 1,343 (56.6) | 603 (61.2) | 1,246 (50.3) | 357 (51.5) |
|  | Missing | 5,628 (21.2) | 771 (27.5) | 285 (26.2) | 747 (22.7) | 234 (26.2) |
| Perceived Access to PPE | Perceived inadequate access | 1,709 (8.3) | 196 (8.7) | 94 (10.3) | 191 (7.0) | 61 (8.5) |
|  | Perceived adequate access | 17,130 (73.8) | 1,767 (67.4) | 700 (68.3) | 2,126 (71.4) | 551 (67.6) |
|  | Non-applicable | 2,839 (8.6) | 199 (6.0) | 72 (5.9) | 404 (10.9) | 100 (11.4) |
|  | Missing | 2,459 (9.3) | 497 (17.9) | 174 (15.5) | 349 (10.7) | 104 (12.5) |
| Confidence in infection control policies | Inadequate confidence | 8,211 (37.7) | 825 (34.6) | 355 (36.1) | 968 (34.2) | 274 (35.7) |
|  | Adequate confidence | 13,406 (53.0) | 1,341 (47.7) | 520 (49.1) | 1,749 (55.3) | 440 (51.5) |
|  | Missing | 2,520 (9.3) | 493 (17.7) | 165 (14.8) | 353 (10.5) | 102 (12.8) |
| n = unweighted frequencies (with weighted percentages); PCS = Post Covid-19 Syndrome (symptoms lasting for 12 weeks following acute Covid-19 infection); NHS = National Health Service in the United Kingdom; NHS Band 5 or below = Annual income of £34,581 or lower; NHS Band 6 or above = Annual income of £35,392 or higher (As of January 2024). | | | | | | |

**Supplemental Table 4c.** Baseline physical health information of the healthcare workers in NHS CHECK, given in terms of the total NHS CHECK cohort and for those who had a previous Covid-19 infection and who reported Post Covid-19 Syndrome for the 12 and 32 month follow-up surveys separately.

|  | | **Total NHS CHECK sample** | **12 Month Follow-Up Survey** | | **32 Month Follow-Up Survey** | |
| --- | --- | --- | --- | --- | --- | --- |
|  |  |  | **Staff with a Previous Covid-19 Infection** | **Staff with PCS** | **Staff with a Previous Covid-19 Infection** | **Staff with PCS** |
| **Physical Health Information** | | ***n* = 24,137 (%)** | ***n* = 2,659 (%)** | ***n* = 1,040 (%)** | ***n* = 3,070 (%)** | ***n* = 816 (%)** |
| Pre-existing respiratory illness | None | 21,338 (89.1) | 2,093 (79.6) | 822 (80.4) | 2,588 (85.9) | 654 (80.6) |
|  | Reported Asthma/COPD | 1,216 (5.0) | 127 (4.4) | 70 (6.2) | 208 (6.0) | 79 (9.6) |
|  | Missing | 1,583 (5.9) | 439 (16.0) | 148 (13.4) | 274 (8.1) | 83 (9.8) |
| Hospitalised during acute Covid-19 infection* | Not hospitalised | – | 2,516 (95.4) | 973 (94.4) | – | – |
|  | Hospitalised | – | 68 (2.1) | 57 (4.8) | – | – |
|  | Missing | – | 75 (2.5) | 10 (0.8) | – | – |
| Covid-19 testing status at time of baseline survey response | Tested, positive result | 1,254 (7.2) | 491 (21.7) | 258 (25.8) | 125 (5.8) | 49 (7.0) |
|  | Tested, negative result | 7,362 (32.0) | 558 (21.3) | 207 (20.0) | 1,012 (35.3) | 278 (35.1) |
|  | Tested, awaiting result | 237 (1.0) | 28 (0.9) | 14 (1.2) | 21 (0.8) | 13 (1.5) |
|  | Tested, inconclusive result | 163 (0.7) | 25 (1.3) | 13 (2.2) | 24 (0.5) | 13 (1.2) |
|  | Never tested | 12,612 (49.7) | 1,069 (37.1) | 378 (35.5) | 1,541 (46.7) | 363 (43.0) |
|  | Missing | 2,509 (9.4) | 488 (17.7) | 170 (15.3) | 347 (10.9) | 100 (12.2) |
| n = unweighted frequencies (with weighted percentages); PCS = Post Covid-19 Syndrome (symptoms lasting for 12 weeks following acute Covid-19 infection); NHS = National Health Service in the United Kingdom; COPD = Chronic Obstructive Pulmonary Disease.  *Hospitalised during acute Covid-19 infection was only measured at 12-months, hence figures for the total NHS CHECK sample and for the 32-month follow-up sample are not presented. | | | | | | |

**Supplemental Table 4d.** Baseline mental health information of the healthcare workers in NHS CHECK, given in terms of the total NHS CHECK cohort and for those who had a previous Covid-19 infection and who reported Post Covid-19 Syndrome for the 12 and 32 month follow-up surveys separately.

| **Mental Health Information** | | **Total NHS CHECK sample** | **12 Month Follow-Up Survey** | | **32 Month Follow-Up Survey** | |
| --- | --- | --- | --- | --- | --- | --- |
|  |  |  | **Staff with a Previous Covid-19 Infection** | **Staff with PCS** | **Staff with a Previous Covid-19 Infection** | **Staff with PCS** |
|  |  | ***n* = 24,137 (%)** | ***n* = 2,659 (%)** | ***n* = 1,040 (%)** | ***n* = 3,070 (%)** | ***n* = 816 (%)** |
| Probable common mental disorders | No (GHQ-12 score <4) | 9,811 (40.9) | 938 (34.6) | 302 (28.6) | 1,197 (38.3) | 237 (25.4) |
|  | Yes (GHQ-12 score ≥4) | 10,970 (45.9) | 1,164 (44.8) | 534 (52.8) | 1,442 (48.3) | 451 (59.1) |
|  | Missing | 3,356 (13.2) | 557 (20.6) | 204 (18.6) | 431 (13.4) | 128 (15.5) |
| Probable depression* | No (PHQ-9 score <10) | 8,694 (35.3) | 1,010 (37.6) | 367 (35.3) | 1,357 (46.1) | 299 (38.6) |
|  | Yes (PHQ-9 score ≥10) | 3,302 (14.3) | 431 (16.2) | 221 (22.4) | 537 (18.5) | 213 (27.4) |
|  | Missing | 12,141 (50.4) | 1,218 (46.2) | 452 (42.3) | 1,176 (35.4) | 304 (34.0) |
| Probable generalized anxiety disorder* | No (GAD-7 score <10) | 9,319 (38.0) | 1,131 (42.0) | 433 (41,7) | 1,500 (51.1) | 365 (47.7) |
|  | Yes (GAD-7 score ≥10) | 2,716 (11.8) | 325 (12.4) | 163 (16.7) | 400 (13.8) | 150 (19.4) |
|  | Missing | 12,102 (50.2) | 1,203 (45.6) | 444 (41.6) | 1,170 (35.1) | 201 (32.9) |
| Probable burnout* | No (BAT-12 score <2.96) | 10,000 (41.1) | 1,197 (44.5) | 469 (44.7) | 1,581 (54.7) | 398 (52.7) |
|  | Yes (BAT-12 score ≥2.96) | 1,820 (7.7) | 230 (8.7) | 113 (12.0) | 299 (9.6) | 112 (14.0) |
|  | Missing | 12,317 (51.2) | 1,232 (46.8) | 458 (43.3) | 1,190 (35.7) | 306 (33.3) |
| Probable post-traumatic stress disorder* | No (PCL-6 score <14) | 9,158 (36.9) | 1,096 (40.6) | 404 (39.1) | 1,455 (48.0) | 351 (42.2) |
|  | Yes (PCL-6 score ≥14) | 2,825 (12.6) | 343 (12.9) | 179 (17.6) | 205 (16.9) | 166 (25.0) |
|  | Missing | 12,154 (50.5) | 1,220 (46.5) | 457 (43.3) | 1,265 (35.1) | 299 (32.8) |
| Probable alcohol use disorder* | No (AUDIT-C score <8) | 10,010 (42.0) | 1,193 (45.4) | 483 (48.1) | 1,600 (54) | 442 (57.1) |
|  | Yes (AUDIT-C score ≥8) | 1,396 (5.3) | 168 (5.7) | 67 (6.1) | 205 (7.3) | 50 (6.4) |
|  | Missing | 12,731 (52.7) | 1,298 (48.9) | 490 (45.8) | 1,265 (38.7) | 324 (36.5) |
| n = unweighted frequencies (with weighted percentages); PCS = Post Covid-19 Syndrome (symptoms lasting for 12 weeks following acute Covid-19 infection); NHS = National Health Service in the United Kingdom; GHQ = General Health Questionnaire; PHQ = Patient Health Questionnaire; GAD = Generalised Anxiety Disorder; BAT = Burnout Assessment Tool; PCL = Post-traumatic stress disorder CheckList; AUDIT-C = Alcohol Use Disorder Identification Test for Consumption. | | | | | | |

*These scales were only completed by those who took part in the longer version of the baseline survey (3,155 of the included 5,248 HCWs), hence the high percentage of missing data.

**Supplemental Table 5a.** Prevalence of Covid-19 symptoms and their duration among HCWs who completed the 12 month follow-up survey (*n* = 2,659).

|  | **No Reported Symptoms** | | **Symptoms for <4 weeks (Acute infection)** | | **Symptoms for ≥4 weeks but <12 weeks (OSC)** | | **Symptoms for ≥12 weeks (PCS)** | | |
| --- | --- | --- | --- | --- | --- | --- | --- | --- | --- |
| **Duration of Any Covid-19 Symptoms** | ***n*** | ***%*** | ***n*** | ***%*** | ***n*** | ***%*** | ***n*** | ***%*** | ***%, PCS only*** |
| Experienced any symptom | 204 | (7.5) | 1,091 | (42.2) | 324 | (12.0) | 1,040 * | (38.3) | - |
| **Duration of Each Covid-19 Symptom** | ***n*** | ***%*** | ***n*** | ***%*** | ***n*** | ***%*** | ***n*** | ***%*** | ***%, PCS only*** |
| Fatigue | 415 | (16.5) | 1,324 | (49.7) | 328 | (12.1) | 592 | (21.7) | (56.4) |
| Difficulty concentrating | 1,491 | (57.1) | 555 | (20.4) | 176 | (6.8) | 437 | (15.7) | (39.8) |
| Anxiety or depression | 1,711 | (64.3) | 485 | (18.1) | 119 | (4.3) | 344 | (13.3) | (34.8) |
| Insomnia | 1,593 | (60.6) | 577 | (21.1) | 135 | (5.2) | 354 | (13.1) | (34.6) |
| Shortness of breath | 1,136 | (43.6) | 970 | (36.4) | 209 | (7.6) | 344 | (12.4) | (3.02) |
| Loss or change in sense of taste/smell | 934 | (36.2) | 1,158 | (43.0) | 232 | (8.6) | 335 | (12.2) | (30.8) |
| Memory loss or confusion | 1,852 | (70.5) | 376 | (14.0) | 113 | (4.0) | 319 | (11.5) | (28.7) |
| Joint pains | 1,044 | (39.1) | 1,222 | (45.8) | 128 | (5.5) | 265 | (9.6) | (25.5) |
| Muscle or body aches | 630 | (23.4) | 1,645 | (62.1) | 145 | (5.5) | 239 | (9.0) | (23.4) |
| Headache | 673 | (24.7) | 1,647 | (62.2) | 151 | (6.1) | 188 | (7.0) | (18.0) |
| Chest pain or tightness | 1,456 | (55.9) | 875 | (32.6) | 140 | (5.0) | 188 | (6.5) | (16.7) |
| Heart palpitations | 1,979 | (73.7) | 422 | (16.5) | 96 | (3.6) | 162 | (6.2) | (16.7) |
| Dizziness | 1,788 | (67.2) | 643 | (24.1) | 95 | (3.6) | 133 | (5.1) | (13.9) |
| Tinnitus | 2,197 | (82.0) | 298 | (11.9) | 23 | (1.0) | 141 | (5.1) | (13.2) |
| Pins and needles or numbness | 2,169 | (81.4) | 310 | (12.4) | 45 | (1.4) | 135 | (4.8) | (13.0) |
| Cough | 966 | (37.3) | 1,379 | (51.3) | 184 | (6.8) | 130 | (4.6) | (12.7) |
| Runny nose or nasal congestion | 1,296 | (48.8) | 1,184 | (44.2) | 84 | (3.3) | 95 | (3.7) | (9.2) |
| Sore throat | 1,303 | (50.1) | 1,236 | (45.2) | 64 | (2.5) | 56 | (2.2) | (5.7) |
| Stomach pain | 2,052 | (76.2) | 514 | (20.3) | 39 | (1.3) | 54 | (2.2) | (5.7) |
| Skin rash | 2,234 | (83.0) | 351 | (14.3) | 24 | (0.9) | 50 | (1.8) | (4.7) |
| Fever, chills or shivering | 861 | (31.2) | 1,718 | (65.3) | 39 | (1.8) | 41 | (1.7) | (4.5) |
| Sneezing | 1,627 | (61.6) | 956 | (35.1) | 33 | (1.4) | 43 | (1.9) | (4.4) |
| Nausea or vomiting | 1,891 | (71.1) | 696 | (26.3) | 31 | (1.0) | 41 | (1.6) | (4.3) |
| Diarrhoea | 1,833 | (68.3) | 756 | (29.3) | 30 | (0.8) | 40 | (1.6) | (4.3) |
| HCWs = Healthcare workers; PCS = Post Covid-19 Syndrome; OSC = Ongoing Symptomatic Covid-19; n = unweighted frequencies; % = percentages weighted based on sex, age, ethnicity and job role of workforce in participating Trusts; %, PCS only = weighted percentages only including staff who met the definition of PCS. *This includes 205 HCWs who reported a formal Long Covid/PCS diagnosis received from a medical professional. | | | | | | | | | |

**Supplemental Table 5b.** Prevalence of Covid-19 symptoms and their duration among HCWs who completed the 32 month follow-up survey (*n* = 3,070).

|  | **No Reported Symptoms** | | **Symptoms for <4 weeks (Acute infection)** | | **Symptoms for ≥4 weeks but <12 weeks (OSC)** | | **Symptoms for ≥12 weeks (PCS)** | | |
| --- | --- | --- | --- | --- | --- | --- | --- | --- | --- |
| **Duration of Any Covid-19 Symptoms** | ***n*** | ***%*** | ***n*** | ***%*** | ***n*** | ***%*** | ***n*** | ***%*** | ***%, PCS only*** |
| Experienced any symptom | 137 | (4.7) | 1,765 | (58.0) | 352 | (11.0) | 816 * | (26.3) | - |
| **Duration of Each Covid-19 Symptom** | ***n*** | ***%*** | ***n*** | ***%*** | ***n*** | ***%*** | ***n*** | ***%*** | ***%, PCS only*** |
| Fatigue | 409 | (14.3) | 1,995 | (65.5) | 321 | (9.0) | 345 | (11.2) | (42.1) |
| Insomnia | 1,642 | (54.2) | 1,008 | (32.8) | 122 | (4.1) | 298 | (8.9) | (33.4) |
| Difficulty concentrating | 1,689 | (56.0) | 958 | (31.0) | 168 | (5.2) | 255 | (7.8) | (29.0) |
| Anxiety or depression | 2,152 | (69.6) | 590 | (20.1) | 105 | (3.4) | 223 | (6.9) | (26.7) |
| Shortness of breath | 1,572 | (51.5) | 1,149 | (37.2) | 156 | (5.1) | 193 | (6.2) | (23.1) |
| Memory loss or confusion | 2,189 | (71.9) | 577 | (18.8) | 107 | (3.4) | 197 | (5.9) | (22.7) |
| Tinnitus | 2.531 | (82.2) | 338 | (12.0) | 25 | (0.7) | 176 | (5.1) | (18.6) |
| Joint pains | 1,221 | (41.7) | 1,599 | (51.0) | 87 | (2.5) | 163 | (4.8) | (18.2) |
| Muscle or body aches | 629 | (21.1) | 2,217 | (72.2) | 89 | (2.4) | 135 | (4.3) | (16.9) |
| Runny nose or nasal congestion | 905 | (31.8) | 1,954 | (62.1) | 89 | (2.4) | 122 | (3.7) | (13.6) |
| Cough | 762 | (26.7) | 1,974 | (63.7) | 203 | (6.0) | 131 | (3.6) | (12.8) |
| Pins and needles or numbness | 2,604 | (83.7) | 328 | (11.8) | 28 | (1.1) | 110 | (3.4) | (12.5) |
| Chest pain or tightness | 1,997 | (65.8) | 878 | (28.2) | 94 | (3.0) | 101 | (3.0) | (11.6) |
| Heart palpitations | 2,435 | (78.2) | 497 | (17.5) | 44 | (1.4) | 94 | (2.9) | (11.0) |
| Loss or change in sense of taste/smell | 1,806 | (61.6) | 1,069 | (32.6) | 98 | (2.8) | 97 | (3.0) | (10.8) |
| Headache | 654 | (23.2) | 2,250 | (71.5) | 72 | (2.4) | 94 | (2.9) | (10.8) |
| Dizziness | 2,143 | (70.4) | 768 | (24.8) | 66 | (2.2) | 93 | (2.6) | (10.1) |
| Sneezing | 1,387 | (46.1) | 1,589 | (51.5) | 43 | (1.1) | 51 | (1.3) | (4.7) |
| Sore throat | 876 | (30.1) | 2,087 | (67.1) | 61 | (1.7) | 46 | (1.1) | (4.1) |
| Stomach pain | 2,480 | (81.1) | 525 | (17.0) | 23 | (0.8) | 42 | (1.1) | (4.1) |
| Diarrhoea | 2,350 | (78.1) | 661 | (20.1) | 28 | (0.8) | 31 | (1.0) | (3.7) |
| Skin rash | 2,720 | (88.4) | 291 | (9.9) | 22 | (0.8) | 37 | (0.9) | (3.5) |
| Nausea or vomiting | 2,358 | (77.9) | 656 | (20.6) | 27 | (0.8) | 29 | (0.7) | (2.9) |
| Fever, chills or shivering | 803 | (26.0) | 2,211 | (72.2) | 31 | (1.0) | 25 | (0.8) | (2.7) |
| HCWs = Healthcare workers; PCS = Post Covid-19 Syndrome; OSC = Ongoing Symptomatic Covid-19; n = unweighted frequencies; % = percentages weighted based on sex, age, ethnicity and job role of workforce in participating Trusts; %, PCS only = weighted percentages only including staff who met the definition of PCS.*This includes 219 HCWs who reported a formal Long Covid/PCS diagnosis received from a medical professional. | | | | | | | | | |

**Supplemental Table 6.** Information on the number of symptoms of PCS reported by the healthcare workers and the number of diagnosed conditions they developed after Covid-19 infection (*n* = 1,730).

|  | ***n*** | ***%*** |
| --- | --- | --- |
| **Number of reported symptoms** |  |  |
| No symptoms* | 60 | (4.0) |
| 1 symptom | 459 | (26.7) |
| 2-3 symptoms | 426 | (26.6) |
| 4-6 symptoms | 392 | (20.6) |
| 7-9 symptoms | 210 | (12.4) |
| 10+ symptoms | 183 | (9.7) |
| **Reported cognitive symptoms**** |  |  |
| Yes | 1,200 | (66.2) |
| No | 530 | (33.8) |
| **Reported general symptoms**** |  |  |
| Yes | 967 | (56.0) |
| No | 763 | (44.0) |
| **Reported mental health problems**** |  |  |
| Yes | 803 | (47.4) |
| No | 927 | (52.6) |
| **Reported cardiopulmonary problems**** |  |  |
| Yes | 799 | (45.1) |
| No | 931 | (54.9) |
| **Reported gestational problems**** |  |  |
| Yes | 154 | (8.7) |
| No | 1,576 | (91.3) |
| **Conditions that developed after Covid-19 infection** | | |
| Formal diagnosis of Long Covid or PCS | 385 | (22.2) |
| Post-viral fatigue syndrome | 134 | (7.2) |
| Anxiety | 140 | (7.0) |
| Depression | 129 | (7.4) |
| Post-traumatic stress disorder | 32 | (2.0) |
| Diabetes (Type 1 or 2) | 132 | (7.0) |
| Heart arrhythmia | 49 | (2.6) |
| Deep-vein thrombosis | 8 | (4.8) |
| Myocardial infarction | 2 | (0.1) |
| Heart failure | 11 | (0.9) |
| Any other heart complication | 27 | (1.3) |
| Pulmonary embolism | 32 | (1.3) |
| Any other lung condition | 45 | (2.3) |
| Kidney disease | 16 | (1.3) |
| Thyroid disease | 27 | (1.6) |
| Any other neurological complication | 19 | (0.9) |
| n = unweighted frequencies; % = weighted percentages.  *These participants did not report any symptoms but did report a doctor diagnosis of Long Covid or PCS (Post- Covid-19 Syndrome; symptoms lasting for 12+ weeks following acute Covid-19 infection)  **Cognitive symptoms (concentration, memory loss, headache, loss or change of taste/smell, dizziness, tinnitus, numbness); General symptoms (Fatigue, muscle ache, joint pains, fever, rash); Mental health symptoms (Depression/anxiety, insomnia); Cardiopulmonary symptoms (Cough, shortness of breath, chest pain, sore throat, heart palpitations, runny nose, sneezing); and Gestational symptoms (nausea, diarrhoea, stomach pain). | | |

**Section C**

**Sensitivity Analyses for the Multi-Level Regression Model Exploring Risk Factors for PCS among HCWs**

**Supplemental Table 7.** Results from the Multi-Level Logistic Regression exploring baseline risk factors for reporting a formal diagnosis of Long Covid or Post Covid-19 Syndrome from a medical professional at least 12 months later among the HCWs included in NHS CHECK (*n* = 5,248).

| **Variable** |  | **Odds of Reporting a Formal Long Covid/PCS Diagnosis** | |
| --- | --- | --- | --- |
|  | **Categories** | **aOR** | **95% CI** |
| Probable common mental disorders | No (GHQ-12 score <10; Ref) | 1 | – |
|  | Yes (GHQ-12 score ≥10) | 1.68** | [1.24, 2.27] |
| Sex | Female (Ref) | 1 | – |
|  | Male | 0.99 | [0.70, 1.40] |
| Age in years | 30 and younger (Ref) | 1 | – |
|  | 31–40 | 1.54 | [0.67, 3.55] |
|  | 41–50 | 1.80* | [1.01, 3.21] |
|  | 51–60 | 1.32 | [0.77, 2.25] |
|  | 61 and older | 0.90 | [0.41, 1.98] |
| Ethnicity | White (Ref) | 1 | – |
|  | Black | 0.25*** | [0.13, 0.48] |
|  | Asian | 0.71 | [0.33, 1.54] |
|  | Mixed/Multiple ethnic group | 0.86 | [0.17, 4.42] |
|  | Other ethnic group | 2.11 | [0.22, 20.36] |
| Job role | Nurse (Ref) | 1 | – |
|  | Doctor | 0.23* | [0.06, 0.96] |
|  | Other clinical | 0.77 | [0.52, 1.16] |
|  | Non-clinical | 0.78 | [0.48, 1.27] |
| Relationship status | Single/Divorced (Ref) | 1 | – |
|  | Married/Cohabitating | 0.72 | [0.45, 1.16] |
| Probable alcohol use disorder | No (AUDIT-C score <8; Ref) | 1 | – |
|  | Yes (AUDIT-C score ≥8) | 0.87 | [0.51, 1.51] |
| Pre-existing respiratory illness | No (Ref) | 1 | – |
|  | Reported Asthma/COPD | 2.93** | [1.44, 5.95] |
| Income | NHS Band 5 or below (Ref) | 1 | – |
|  | NHS Band 6 or above | 0.80 | [0.54, 1.18] |
| Contact with Covid-19 patients | No (Ref) | 1 | – |
|  | Yes | 1.63* | [1.03, 2.59] |
| Perceived access to PPE | Perceived inadequate access (Ref) | 1 | – |
|  | Perceived adequate access | 0.87 | [0.48, 1.57] |
|  | Non-applicable | 1.51 | [0.76, 3.00] |
| Confidence in infection control policies | Inadequate confidence (Ref) | 1 | – |
|  | Adequate confidence | 1.03 | [0.73, 1.44] |
| HCWs = Healthcare workers; PCS = Post Covid-19 Syndrome (symptoms lasting for 12 or more weeks following acute Covid-19 infection); aOR = Adjusted Odds Ratio, adjusted for all variables in the table, as well as burden on NHS when risk factor data were collected and Trust; 95% CI = 95% Confidence Intervals; GHQ = General Health Questionnaire; COPD = Chronic Obstructive Pulmonary Disease; NHS = National Health Service in the United Kingdom; NHS Band 5 or below = Annual income of £34,581 or lower; NHS Band 6 or above = Annual income of £35,392 or higher (As of January 2024). * *p* < 0.05; ** *p* ≤ 0.01; *** *p* ≤ 0.001. | | | |

**Supplemental Table 8.** Results from the Multi-Level Logistic Regression exploring baseline risk factors for reporting Long Covid (using the broad NICE definition of symptoms lasting for 4 or more weeks) at least 12 months later among the HCWs included in NHS CHECK (*n* = 5,248).

| **Variable** |  | **Odds of Reporting LC** | |
| --- | --- | --- | --- |
|  | **Categories** | **aOR** | **95% CI** |
| Probable common mental disorders | No (GHQ-12 score <10; Ref) | 1 | – |
|  | Yes (GHQ-12 score ≥10) | 1.80*** | [1.44, 2.26] |
| Sex | Female (Ref) | 1 | – |
|  | Male | 0.69*** | [0.58, 0.83] |
| Age in years | 30 and younger (Ref) | 1 | – |
|  | 31–40 | 1.33 | [0.88, 2.02] |
|  | 41–50 | 1.56 | [0.99, 2.44] |
|  | 51–60 | 1.42* | [1.08, 1.88] |
|  | 61 and older | 1.10 | [0.72, 1.68] |
| Ethnicity | White (Ref) | 1 | – |
|  | Black | 0.77 | [0.40, 1.48] |
|  | Asian | 0.89 | [0.50, 1.57] |
|  | Mixed/Multiple ethnic group | 0.75 | [0.38, 1.49] |
|  | Other ethnic group | 1.14 | [0.50, 2.57] |
| Job role | Nurse (Ref) | 1 | – |
|  | Doctor | 0.77 | [0.55, 1.09] |
|  | Other clinical | 0.72* | [0.54, 0.98] |
|  | Non-clinical | 0.84 | [0.63, 1.13] |
| Relationship status | Single/Divorced (Ref) | 1 | – |
|  | Married/Cohabitating | 1.00 | [0.84, 1.19] |
| Probable alcohol use disorder | No (AUDIT-C score <8; Ref) | 1 | – |
|  | Yes (AUDIT-C score ≥8) | 1.19 | [0.88, 1.62] |
| Pre-existing respiratory illness | No (Ref) | 1 | – |
|  | Reported Asthma/COPD | 1.51* | [1.10, 2.07] |
| Income | NHS Band 5 or below (Ref) | 1 | – |
|  | NHS Band 6 or above | 0.85 | [0.61, 1.17] |
| Contact with Covid-19 patients | No (Ref) | 1 | – |
|  | Yes | 1.60*** | [1.29, 1.99] |
| Perceived access to PPE | Perceived inadequate access (Ref) | 1 | – |
|  | Perceived adequate access | 0.74 | [0.46, 1.22] |
|  | Non-applicable | 0.88 | [0.50, 1.54] |
| Confidence in infection control policies | Inadequate confidence (Ref) | 1 | – |
|  | Adequate confidence | 0.88 | [0.76, 1.03] |
| HCWs = Healthcare workers; LC = Long Covid (symptoms lasting for 4 or more weeks following acute Covid-19 infection); aOR = Adjusted Odds Ratio, adjusted for all variables in the table, as well as burden on NHS when risk factor data were collected and Trust; 95% CI = 95% Confidence Intervals; GHQ = General Health Questionnaire; COPD = Chronic Obstructive Pulmonary Disease; NHS = National Health Service in the United Kingdom; NHS Band 5 or below = Annual income of £34,581 or lower; NHS Band 6 or above = Annual income of £35,392 or higher (As of January 2024). * *p* < 0.05; *** *p* ≤ 0.001. | | | |

**Supplemental Table 9a.** Results from the Multi-Level Logistic Regression exploring baseline risk factors for reporting Post Covid-19 Syndrome at least 12 months later among the HCWs included in NHS CHECK, using only data collected by the 12 month follow-up survey (*n* = 2,659).

| **Variable** |  | **Odds of Reporting PCS** | |
| --- | --- | --- | --- |
|  | **Categories** | **aOR** | **95% CI** |
| Probable common mental disorders | No (GHQ-12 score <10; Ref) | 1 | – |
|  | Yes (GHQ-12 score ≥10) | 1.92*** | [1.54, 2.40] |
| Sex | Female (Ref) | 1 | – |
|  | Male | 0.74* | [0.59, 0.93] |
| Age in years | 30 and younger (Ref) | 1 | – |
|  | 31–40 | 1.77 | [0.72, 4.41] |
|  | 41–50 | 2.26* | [1.05, 4.88] |
|  | 51–60 | 2.07*** | [1.42, 3.02] |
|  | 61 and older | 1.43 | [0.79, 2.58] |
| Ethnicity | White (Ref) | 1 | – |
|  | Black | 0.63 | [0.27, 1.47] |
|  | Asian | 0.84 | [0.44, 1.63] |
|  | Mixed/Multiple ethnic group | 1.06 | [0.52, 2.18] |
|  | Other ethnic group | 0.80 | [0.26, 2.51] |
| Job role | Nurse (Ref) | 1 | – |
|  | Doctor | 0.64 | [0.38, 1.07] |
|  | Other clinical | 0.81 | [0.49, 1.34] |
|  | Non-clinical | 0.85 | [0.53, 1.35] |
| Relationship status | Single/Divorced (Ref) | 1 | – |
|  | Married/Cohabitating | 1.00 | [0.73, 1.38] |
| Probable alcohol use disorder | No (AUDIT-C score <8; Ref) | 1 | – |
|  | Yes (AUDIT-C score ≥8) | 1.03 | [0.69, 1.53] |
| Pre-existing respiratory illness | No (Ref) | 1 | – |
|  | Reported Asthma/COPD | 1.37 | [0.79, 2.38] |
| Income | NHS Band 5 or below (Ref) | 1 | – |
|  | NHS Band 6 or above | 0.89 | [0.79, 2.38] |
| Contact with Covid-19 patients | No (Ref) | 1 | – |
|  | Yes | 1.61* | [1.02, 2.55] |
| Perceived access to PPE | Perceived inadequate access (Ref) | 1 | – |
|  | Perceived adequate access | 0.59 | [0.24, 1.43] |
|  | Non-applicable | 0.67 | [0.24, 1.88] |
| Confidence in infection control policies | Inadequate confidence (Ref) | 1 | – |
|  | Adequate confidence | 1.05 | [0.79, 1.39] |
| HCWs = Healthcare workers; PCS = Post Covid-19 Syndrome (symptoms lasting for 12 or more weeks following acute Covid-19 infection); aOR = Adjusted Odds Ratio, adjusted for all variables in the table, as well as burden on NHS when risk factor data were collected and Trust; 95% CI = 95% Confidence Intervals; GHQ = General Health Questionnaire; COPD = Chronic Obstructive Pulmonary Disease; NHS = National Health Service in the United Kingdom; NHS Band 5 or below = Annual income of £34,581 or lower; NHS Band 6 or above = Annual income of £35,392 or higher (As of January 2024). * *p* < 0.05; *** *p* ≤ 0.001. | | | |

**Supplemental Table 9b.** Results from the Multi-Level Logistic Regression exploring baseline risk factors for reporting Post Covid-19 Syndrome at least 12 months later among the HCWs included in NHS CHECK, including hospitalisation as a risk factor and using only data collected by the 12 month follow-up survey (*n* = 2,584).

| **Variable** |  | **Odds of Reporting PCS** | |
| --- | --- | --- | --- |
|  | **Categories** | **aOR** | **95% CI** |
| Probable common mental disorders | No (GHQ-12 score <10; Ref) | 1 | – |
|  | Yes (GHQ-12 score ≥10) | 1.90*** | [1.52, 2.37] |
| Sex | Female (Ref) | 1 | – |
|  | Male | 0.73* | [0.57, 0.93] |
| Age in years | 30 and younger (Ref) | 1 | – |
|  | 31–40 | 1.73 | [0.69, 4.37] |
|  | 41–50 | 2.24* | [1.08, 4.65] |
|  | 51–60 | 2.05*** | [1.42, 2.94] |
|  | 61 and older | 1.35 | [0.75, 2.43] |
| Ethnicity | White (Ref) | 1 | – |
|  | Black | 0.64 | [0.27, 1.50] |
|  | Asian | 0.78 | [0.41, 1.46] |
|  | Mixed/Multiple ethnic group | 0.98 | [0.50, 1.94] |
|  | Other ethnic group | 0.80 | [0.26, 2.49] |
| Job role | Nurse (Ref) | 1 | – |
|  | Doctor | 0.60 | [0.36, 1.01] |
|  | Other clinical | 0.83 | [0.50, 1.37] |
|  | Non-clinical | 0.86 | [0.53, 1.39] |
| Relationship status | Single/Divorced (Ref) | 1 | – |
|  | Married/Cohabitating | 1.02 | [0.75, 1.38] |
| Probable alcohol use disorder | No (AUDIT-C score <8; Ref) | 1 | – |
|  | Yes (AUDIT-C score ≥8) | 1.04 | [0.69, 1.57] |
| Pre-existing respiratory illness | No (Ref) | 1 | – |
|  | Reported Asthma/COPD | 1.27 | [0.73, 2.21] |
| Hospitalised during acute infection | No (Ref) | 1 | – |
|  | Yes | 8.29*** | [4.12, 16.70] |
| Income | NHS Band 5 or below (Ref) | 1 | – |
|  | NHS Band 6 or above | 0.91 | [0.63, 1.30] |
| Contact with Covid-19 patients | No (Ref) | 1 | – |
|  | Yes | 1.58 | [0.99, 2.52] |
| Perceived access to PPE | Perceived inadequate access (Ref) | 1 | – |
|  | Perceived adequate access | 0.55 | [0.22, 1.39] |
|  | Non-applicable | 0.63 | [0.21, 1.84] |
| Confidence in infection control policies | Inadequate confidence (Ref) | 1 | – |
|  | Adequate confidence | 1.07 | [0.80, 1.42] |
| HCWs = Healthcare workers; PCS = Post Covid-19 Syndrome (symptoms lasting for 12 or more weeks following acute Covid-19 infection); aOR = Adjusted Odds Ratio, adjusted for all variables in the table, as well as burden on NHS when risk factor data were collected and Trust; 95% CI = 95% Confidence Intervals; GHQ = General Health Questionnaire; COPD = Chronic Obstructive Pulmonary Disease; NHS = National Health Service in the United Kingdom; NHS Band 5 or below = Annual income of £34,581 or lower; NHS Band 6 or above = Annual income of £35,392 or higher (As of January 2024). * *p* < 0.05; *** *p* ≤ 0.001.  Note. Hospitalisation during acute Covid-19 infection was not included in the multiple imputation using chained equations imputation model, hence a decrease in the number of participants. | | | |

**Supplemental Table 9c.** Results from the Multi-Level Logistic Regression exploring baseline risk factors for reporting Post Covid-19 Syndrome at least 12 months later among the HCWs included in NHS CHECK, using only data collected by the 32 month follow-up survey (*n* = 3,070).

| **Variable** |  | **Odds of Reporting PCS** | |
| --- | --- | --- | --- |
|  | **Categories** | **aOR** | **95% CI** |
| Probable common mental disorders | No (GHQ-12 score <10; Ref) | 1 | – |
|  | Yes (GHQ-12 score ≥10) | 2.01*** | [1.56, 2.59] |
| Sex | Female (Ref) | 1 | – |
|  | Male | 0.87 | [0.62, 1.22] |
| Age in years | 30 and younger (Ref) | 1 | – |
|  | 31–40 | 1.55 | [0.95, 2.52] |
|  | 41–50 | 1.59 | [0.97, 2.59] |
|  | 51–60 | 1.86** | [1.21, 2.84] |
|  | 61 and older | 1.98 | [1.00, 3.92] |
| Ethnicity | White (Ref) | 1 | – |
|  | Black | 0.66 | [0.22, 1.97] |
|  | Asian | 0.61 | [0.23, 1.58] |
|  | Mixed/Multiple ethnic group | 0.58 | [0.23, 1.47] |
|  | Other ethnic group | 1.49 | [0.49, 4.58] |
| Job role | Nurse (Ref) | 1 | – |
|  | Doctor | 0.44* | [0.23, 0.83] |
|  | Other clinical | 0.75* | [0.57, 0.99] |
|  | Non-clinical | 0.88 | [0.68, 1.15] |
| Relationship status | Single/Divorced (Ref) | 1 | – |
|  | Married/Cohabitating | 1.04 | [0.78, 1.39] |
| Probable alcohol use disorder | No (AUDIT-C score <8; Ref) | 1 | – |
|  | Yes (AUDIT-C score ≥8) | 0.83 | [0.48, 1.45] |
| Pre-existing respiratory illness | No (Ref) | 1 | – |
|  | Reported Asthma/COPD | 2.17* | [1.14, 3.92] |
| Income | NHS Band 5 or below (Ref) | 1 | – |
|  | NHS Band 6 or above | 0.74 | [0.49, 1.12] |
| Contact with Covid-19 patients | No (Ref) | 1 | – |
|  | Yes | 1.59** | [1.18, 2.13] |
| Perceived access to PPE | Perceived inadequate access (Ref) | 1 | – |
|  | Perceived adequate access | 0.83 | [0.51, 1.34] |
|  | Non-applicable | 1.04 | [0.60, 1.79] |
| Confidence in infection control policies | Inadequate confidence (Ref) | 1 | – |
|  | Adequate confidence | 0.87 | [0.63, 1.20] |
| HCWs = Healthcare workers; PCS = Post Covid-19 Syndrome (symptoms lasting for 12 or more weeks following acute Covid-19 infection); aOR = Adjusted Odds Ratio, adjusted for all variables in the table, as well as burden on NHS when risk factor data were collected and Trust; 95% CI = 95% Confidence Intervals; GHQ = General Health Questionnaire; COPD = Chronic Obstructive Pulmonary Disease; NHS = National Health Service in the United Kingdom; NHS Band 5 or below = Annual income of £34,581 or lower; NHS Band 6 or above = Annual income of £35,392 or higher (As of January 2024). * *p* < 0.05; ** *p* ≤ 0.01; *** *p* ≤ 0.001. | | | |

**Supplemental Table 10a.** Results from the Multi-Level Logistic Regression exploring baseline risk factors for reporting Post Covid-19 Syndrome at least 12 months later among the HCWs included in NHS CHECK, using probable depression (measured by the PHQ-9) as the mental disorder variable (*n* = 5,248).

| **Baseline Variables** |  | **Odds of Reporting PCS** | |
| --- | --- | --- | --- |
|  | **Categories** | **aOR** | **95% CI** |
| Probable depression | No (PHQ-9 score <10; Ref) | 1 | – |
|  | Yes (PHQ-9 score ≥10) | 1.93*** | [1.55, 2.39] |
| Sex | Female (Ref) | 1 | – |
|  | Male | 0.78** | [0.66, 0.93] |
| Age in years | 30 and younger (Ref) | 1 | – |
|  | 31–40 | 1.55 | [0.84, 2.87] |
|  | 41–50 | 1.80 | [0.98, 3.32] |
|  | 51–60 | 1.92*** | [1.39, 2.64] |
|  | 61 and older | 1.52 | [0.92, 2.50] |
| Ethnicity | White (Ref) | 1 | – |
|  | Black | 0.75 | [0.31, 1.83] |
|  | Asian | 0.70 | [0.35, 1.39] |
|  | Mixed/Multiple ethnic group | 0.94 | [0.47, 1.90] |
|  | Other ethnic group | 1.13 | [0.46, 2.76] |
| Job role | Nurse (Ref) | 1 | – |
|  | Doctor | 0.65 | [0.41, 1.02] |
|  | Other clinical | 0.76 | [0.57, 1.02] |
|  | Non-clinical | 0.78* | [0.61, 0.99] |
| Relationship status | Single/Divorced (Ref) | 1 | – |
|  | Married/Cohabitating | 1.07 | [0.84, 1.36] |
| Probable alcohol use disorder | No (AUDIT-C score <8; Ref) | 1 | – |
|  | Yes (AUDIT-C score ≥8) | 0.94 | [0.67, 1.31] |
| Pre-existing respiratory illness | No (Ref) | 1 | – |
|  | Reported asthma/COPD | 1.53* | [1.12, 2.10] |
| Income | NHS Band 5 or below (Ref) | 1 | – |
|  | NHS Band 6 or above | 0.86 | [0.64, 1.16] |
| Contact with Covid-19 patients | No (Ref) | 1 | – |
|  | Yes | 1.70*** | [1.30, 2.24] |
| Perceived access to personal protective equipment | Perceived inadequate access (Ref) | 1 | – |
|  | Perceived adequate access | 0.59 | [0.33, 1.07] |
|  | Non-applicable | 0.73 | [0.37, 1.44] |
| Confidence in infection control policies | Inadequate confidence (Ref) | 1 | – |
|  | Adequate confidence | 0.97 | [0.82, 1.15] |
| HCWs **=** Healthcare workers; PCS = Post Covid-19 Syndrome (symptoms lasting for 12+ weeks following acute Covid-19 infection); aOR = Adjusted Odds Ratio, adjusted for all variables in the table, as well as burden on NHS when risk factor data were collected and Trust; 95% CI = 95% Confidence Intervals; COPD = Chronic Obstructive Pulmonary Disease; NHS = National Health Service in the United Kingdom; NHS Band 5 or below = Annual income of £34,581 or lower; NHS Band 6 or above = Annual income of £35,392 or higher (As of January 2024). * *p* < 0.05; ** *p* ≤ 0.01; *** *p* ≤ 0.001. | | | |

**Supplemental Table 10b.** Results from the Multi-Level Logistic Regression exploring baseline risk factors for reporting Post Covid-19 Syndrome at least 12 months later among the HCWs included in NHS CHECK, using probable generalized anxiety disorder (measured by the GAD-7) as the mental disorder variable (*n* = 5,248).

| **Variable** |  | **Odds of Reporting PCS** | |
| --- | --- | --- | --- |
|  | **Categories** | **aOR** | **95% CI** |
| Probable generalized anxiety disorder | No (GAD-7 score <10; Ref) | 1 | – |
|  | Yes (GAD-7 score ≥10) | 1.68*** | [1.33, 2.12] |
| Sex | Female (Ref) | 1 | – |
|  | Male | 0.78** | [0.66, 0.92] |
| Age in years | 30 and younger (Ref) | 1 | – |
|  | 31–40 | 1.54 | [0.83, 2.87] |
|  | 41–50 | 1.77 | [0.93, 3.36] |
|  | 51–60 | 1.87** | [1.30, 2.69] |
|  | 61 and older | 1.44 | [0.89, 2.33] |
| Ethnicity | White (Ref) | 1 | – |
|  | Black | 0.72 | [0.30, 1.73] |
|  | Asian | 0.70 | [0.35, 1.41] |
|  | Mixed/Multiple ethnic group | 0.87 | [0.40, 1.91] |
|  | Other ethnic group | 1.10 | [0.45, 2.65] |
| Job role | Nurse (Ref) | 1 | – |
|  | Doctor | 0.66 | [0.42, 1.05] |
|  | Other clinical | 0.77 | [0.57, 1.03] |
|  | Non-clinical | 0.78 | [0.61, 1.01] |
| Relationship status | Single/Divorced (Ref) | 1 | – |
|  | Married/Cohabitating | 1.04 | [0.83, 1.30] |
| Probable alcohol use disorder | No (AUDIT-C score <8; Ref) | 1 | – |
|  | Yes (AUDIT-C score ≥8) | 0.95 | [0.67, 1.33] |
| Pre-existing respiratory illness | No (Ref) | 1 | – |
|  | Reported Asthma/COPD | 1.57** | [1.15, 2.15] |
| Income | NHS Band 5 or below (Ref) | 1 | – |
|  | NHS Band 6 or above | 0.85 | [0.63, 1.13] |
| Contact with Covid-19 patients | No (Ref) | 1 | – |
|  | Yes | 1.71*** | [1.30, 2.25] |
| Perceived access to PPE | Perceived inadequate access (Ref) | 1 | – |
|  | Perceived adequate access | 0.58 | [0.32, 1.05] |
|  | Non-applicable | 0.71 | [0.36, 1.43] |
| Confidence in infection control policies | Inadequate confidence (Ref) | 1 | – |
|  | Adequate confidence | 0.95 | [0.80, 1.13] |
| HCWs = Healthcare workers; PCS = Post Covid-19 Syndrome (symptoms lasting for 12 or more weeks following acute Covid-19 infection); aOR = Adjusted Odds Ratio, adjusted for all variables in the table, as well as burden on NHS when risk factor data were collected and Trust; 95% CI = 95% Confidence Intervals; COPD = Chronic Obstructive Pulmonary Disease; NHS = National Health Service in the United Kingdom; NHS Band 5 or below = Annual income of £34,581 or lower; NHS Band 6 or above = Annual income of £35,392 or higher (As of January 2024). ** *p* ≤ 0.01; *** *p* ≤ 0.001. | | | |

**Supplemental Table 10c.** Results from the Multi-Level Logistic Regression exploring baseline risk factors for reporting Post Covid-19 Syndrome at least 12 months later among the HCWs included in NHS CHECK, using probable burnout (measured by the BAT-12) as the mental disorder variable (*n* = 5,248).

| **Variable** |  | **Odds of Reporting PCS** | |
| --- | --- | --- | --- |
|  | **Categories** | **aOR** | **95% CI** |
| Probable burnout | No (BAT-12 score <2.96; Ref) | 1 | – |
|  | Yes (BAT-12 score ≥2.96) | 1.58*** | [1.16, 2.15] |
| Sex | Female (Ref) | 1 | – |
|  | Male | 0.77** | [0.66, 0.90] |
| Age in years | 30 and younger (Ref) | 1 | – |
|  | 31–40 | 1.53 | [0.81, 2.87] |
|  | 41–50 | 1.73 | [0.90, 3.33] |
|  | 51–60 | 1.81** | [1.27, 2.58] |
|  | 61 and older | 1.38 | [0.87, 2.20] |
| Ethnicity | White (Ref) | 1 | – |
|  | Black | 0.76 | [0.31, 1.83] |
|  | Asian | 0.69 | [0.36, 1.34] |
|  | Mixed/Multiple ethnic group | 0.89 | [0.44, 1.83] |
|  | Other ethnic group | 1.14 | [0.46, 2.78] |
| Job role | Nurse (Ref) | 1 | – |
|  | Doctor | 0.64* | [0.41, 0.99] |
|  | Other clinical | 0.75 | [0.55, 1.01] |
|  | Non-clinical | 0.78 | [0.60, 1.02] |
| Relationship status | Single/Divorced (Ref) | 1 | – |
|  | Married/Cohabitating | 1.04 | [0.84, 1.29] |
| Probable alcohol use disorder | No (AUDIT-C score <8; Ref) | 1 | – |
|  | Yes (AUDIT-C score ≥8) | 0.97 | [0.70, 1.35] |
| Pre-existing respiratory illness | No (Ref) | 1 | – |
|  | Reported Asthma/COPD | 1.57** | [1.15, 2.15] |
| Income | NHS Band 5 or below (Ref) | 1 | – |
|  | NHS Band 6 or above | 0.82 | [0.61, 1.10] |
| Contact with Covid-19 patients | No (Ref) | 1 | – |
|  | Yes | 1.74*** | [1.33, 2.27] |
| Perceived access to PPE | Perceived inadequate access (Ref) | 1 | – |
|  | Perceived adequate access | 0.59 | [0.33, 1.07] |
|  | Non-applicable | 0.72 | [0.37, 1.44] |
| Confidence in infection control policies | Inadequate confidence (Ref) | 1 | – |
|  | Adequate confidence | 0.96 | [0.81, 1.14] |
| HCWs = Healthcare workers; PCS = Post Covid-19 Syndrome (symptoms lasting for 12 or more weeks following acute Covid-19 infection); aOR = Adjusted Odds Ratio, adjusted for all variables in the table, as well as burden on NHS when risk factor data were collected and Trust; 95% CI = 95% Confidence Intervals; COPD = Chronic Obstructive Pulmonary Disease; NHS = National Health Service in the United Kingdom; NHS Band 5 or below = Annual income of £34,581 or lower; NHS Band 6 or above = Annual income of £35,392 or higher (As of January 2024). * *p* < 0.05; ** *p* ≤ 0.01; *** *p* ≤ 0.001. | | | |

**Supplemental Table 10d.** Results from the Multi-Level Logistic Regression exploring baseline risk factors for reporting Post Covid-19 Syndrome at least 12 months later among the HCWs included in NHS CHECK, using probable post-traumatic stress disorder (measured by the PCL-6) as the mental disorder variable (*n* = 5,248).

| **Variable** |  | **Odds of Reporting PCS** | |
| --- | --- | --- | --- |
|  | **Categories** | **aOR** | **95% CI** |
| Probable post-traumatic stress disorder | No (PCL-6 score <14; Ref) | 1 | – |
|  | Yes (PCL-6 score ≥14) | 1.84*** | [1.40, 2.42] |
| Sex | Female (Ref) | 1 | – |
|  | Male | 0.79** | [0.67, 0.93] |
| Age in years | 30 and younger (Ref) | 1 | – |
|  | 31–40 | 1.51 | [0.82, 2.80] |
|  | 41–50 | 1.75 | [0.91, 3.36] |
|  | 51–60 | 1.81** | [1.29, 2.54] |
|  | 61 and older | 1.42 | [0.92, 2.21] |
| Ethnicity | White (Ref) | 1 | – |
|  | Black | 0.75 | [0.31, 1.82] |
|  | Asian | 0.67 | [0.33, 1.34] |
|  | Mixed/Multiple ethnic group | 0.81 | [0.37, 1.76] |
|  | Other ethnic group | 1.30 | [0.49, 3.44] |
| Job role | Nurse (Ref) | 1 | – |
|  | Doctor | 0.63* | [0.41, 0.98] |
|  | Other clinical | 0.74 | [0.55, 1.01] |
|  | Non-clinical | 0.77 | [0.58, 1.01] |
| Relationship status | Single/Divorced (Ref) | 1 | – |
|  | Married/Cohabitating | 1.07 | [0.87, 1.32] |
| Probable alcohol use disorder | No (AUDIT-C score <8; Ref) | 1 | – |
|  | Yes (AUDIT-C score ≥8) | 0.92 | [0.65, 1.30] |
| Pre-existing respiratory illness | No (Ref) | 1 | – |
|  | Reported Asthma/COPD | 1.50* | [1.11, 2.03] |
| Income | NHS Band 5 or below (Ref) | 1 | – |
|  | NHS Band 6 or above | 0.83 | [0.62, 1.12] |
| Contact with Covid-19 patients | No (Ref) | 1 | – |
|  | Yes | 1.70*** | [1.29, 2.24] |
| Perceived access to PPE | Perceived inadequate access (Ref) | 1 | – |
|  | Perceived adequate access | 0.58 | [0.32, 1.04] |
|  | Non-applicable | 0.71 | [0.36, 1.39] |
| Confidence in infection control policies | Inadequate confidence (Ref) | 1 | – |
|  | Adequate confidence | 0.95 | [0.81, 1.13] |
| HCWs = Healthcare workers; PCS = Post Covid-19 Syndrome (symptoms lasting for 12 or more weeks following acute Covid-19 infection); aOR = Adjusted Odds Ratio, adjusted for all variables in the table, as well as burden on NHS when risk factor data were collected and Trust; 95% CI = 95% Confidence Intervals; COPD = Chronic Obstructive Pulmonary Disease; NHS = National Health Service in the United Kingdom; NHS Band 5 or below = Annual income of £34,581 or lower; NHS Band 6 or above = Annual income of £35,392 or higher (As of January 2024). * *p* < 0.05; ** *p* ≤ 0.01; *** *p* ≤ 0.001. | | | |
